# Role of phytochromes A and B in the regulation of cell death and acclimatory responses to UV stress in *Arabidopsis thaliana*

**DOI:** 10.1093/jxb/erv375

**Published:** 2015-09-18

**Authors:** Anna Rusaczonek, Weronika Czarnocka, Sylwia Kacprzak, Damian Witoń, Ireneusz Ślesak, Magdalena Szechyńska-Hebda, Piotr Gawroński, Stanisław Karpiński

**Affiliations:** ^1^Department of Plant Genetics, Breeding and Biotechnology, Faculty of Horticulture, Biotechnology and Landscape Architecture, Warsaw University of Life Sciences (SGGW), Nowoursynowska Street 159, Warsaw, 02-776Poland; ^2^Department of Botany, Faculty of Agriculture and Biology, Warsaw University of Life Sciences (SGGW), Nowoursynowska Street 159, 02-776 Warsaw, Poland; ^3^The Franciszek Górski Institute of Plant Physiology, Polish Academy of Sciences, Niezapominajek Street 21, 30–239 Krakow, Poland

**Keywords:** Antioxidant enzymes, cell death, photosynthesis, phytochromes, reactive oxygen species, UV stress.

## Abstract

Plants coordinate their responses to various biotic and abiotic stresses in order to optimize their developmental and acclimatory programmes. The ultimate response to an excessive amount of stress is local induction of cell death mechanisms. The death of certain cells can help to maintain tissue homeostasis and enable nutrient remobilization, thus increasing the survival chances of the whole organism in unfavourable environmental conditions. UV radiation is one of the environmental factors that negatively affects the photosynthetic process and triggers cell death. The aim of this work was to evaluate a possible role of the red/far-red light photoreceptors phytochrome A (phyA) and phytochrome B (phyB) and their interrelations during acclimatory responses to UV stress. We showed that UV-C treatment caused a disturbance in photosystem II and a deregulation of photosynthetic pigment content and antioxidant enzymes activities, followed by increased cell mortality rate in *phyB* and *phyAB* null mutants. We also propose a regulatory role of phyA and phyB in CO_2_ assimilation, non-photochemical quenching, reactive oxygen species accumulation and salicylic acid content. Taken together, our results suggest a novel role of phytochromes as putative regulators of cell death and acclimatory responses to UV.

## Highlight

Phytochromes A and B are complex regulators of photosynthesis, reactive oxygen species and salicylic acid homeostasis, and UV-C-induced programmed cell death in *Arabidopsis thaliana.*

## Introduction

In the natural environment, plant growth and development are challenged by ceaseless fluctuations of numerous factors. Plants, being exposed to diverse biotic and abiotic stresses, have developed the ability to perceive and react to both sudden and seasonal changes. One of the most unstable abiotic factors is light. In the course of evolution, plants have formed active mechanisms of light perception that are dependent on photoreceptors (Karpiński *et al.*, 2003; Chen *et al.*, 2004; Möglich *et al.*, 2010; Chen and Chory, 2011) and photosystems (Szechyńska-Hebda *et al.*, 2010). These mechanisms allow plants to sense changes in both, the spectrum and intensity of light. Deciphering the light spectral composition enables plants to regulate the processes associated with their growth and development.

The most intensively studied plant photoreceptors are phytochromes, which are able to absorb red and far-red light. In *Arabidopsis thaliana*, they are represented by five isoforms: light-labile phyA and light-stable phyB–phyE (Quail, 2002). Phytochromes are synthesized in the cytoplasm as inactive precursor forms (Pr). The red light absorption triggers their conversion into active forms (Pfr) and subsequent transfer to the nucleus (Schäfer and Bowler, 2002). Photoactivated phytochromes inactivate phytochrome-interacting transcription factors (PIFs), which inhibit light signalling (Khanna *et al.*, 2004; Jeong and Choi, 2013). By direct interaction with PIFs, phytochromes induce their detachment from DNA and promote their degradation by the 26S proteasome (Park *et al*., 2004, 2012).

Phytochromes regulate every step of plant development and control many diverse physiological processes. Two of them, phyA and phyB, play the principal roles. They control chlorophyll synthesis and de-etiolation (McCormac and Terry, 2002), antagonistically mediate shade-avoidance responses (Casal, 2013; Trupkin *et al.*, 2014) and flowering (Halliday *et al.*, 1994; Franklin and Quail, 2010), and positively regulate stomatal development (Casson *et al.*, 2009). Apart from controlling plant photomorphogenesis, phytochromes have been linked to stress signalling pathways. Using *Arabidopsis* phytochrome-deficient mutants and microarray analysis, Franklin and Whitelam (2007) showed that at low temperature they affect the transcription of Cold Regulated (COR) genes from the C-repeat-binding-factor (CBF) family. Downregulation of these genes is controlled by phyB and phyD, and suggests that phytochromes play a role in freezing tolerance. Other transcription factors of the DRE-binding (DREB) family, involved in freezing responses and dehydration stress, are also regulated indirectly by phytochromes (Kidokoro *et al.*, 2009). The ability of phytochromes to influence plant water status (Casson *et al.*, 2009) and carbon metabolism (Boccalandro *et al.*, 2009) indicates that phytochromes are also implicated in drought stress responses. phyA, phyB, and phyE were proposed to cooperate in the control of drought stress by the regulation of stomatal conductance and abscisic acid (ABA) levels (Boggs *et al.*, 2010). Furthermore, it has been demonstrated that phytochromes can coordinate pathogen defence responses, participating in cross-talk between salicylic acid (SA), protein phosphatase 7 (PP7) and nucleotide-diphosphate kinase 2 (NDPK2) (Genoud *et al.*, 2008).

Programmed cell death (PCD) is one of the most important processes regulating cell fate and thus plant growth and development. Differentiation of some tissues such as tracheary elements involves the selective elimination of viable cells. PCD is induced during various biotic and abiotic stresses (Chichkova *et al.*, 2004; de Pinto *et al.*, 2012). Under stress conditions, plants overproduce reactive oxygen species (ROS), which disturb cellular homeostasis and trigger cell death. On the other hand, PCD helps to maintain tissue homeostasis and enables nutrient remobilization from dying cells, thus increasing the probability of survival in adverse conditions. At the biochemical and molecular levels, plants and animals share some characteristic steps in the PCD programme, such as DNA fragmentation (Kuthanova *et al.*, 2008), chromatin condensation (McCabe *et al.*, 1997), and caspase-like proteolysis (Chichkova *et al.*, 2004). However, in contrast to animals, plants possess chloroplasts in which the imbalances within photosynthetic electron transport can trigger PCD signalling pathways (Samuilov *et al.*, 2003).

LESION SIMULATING DISEASE1 (LSD1) has been described to integrate signalling pathways in response to diverse stresses, both biotic (Rustérucci *et al.*, 2001) and abiotic (Mateo *et al.*, 2004; Mühlenbock *et al.*, 2007, 2008; Wituszyńska *et al.*, 2013b). The LSD1 mutant (*lsd1*) is one of the best-characterized *Arabidopsis* mutants in the context of deregulated cell death. It demonstrates uncontrolled spread of PCD that develops under non-permissive conditions, such as infection with avirulent pathogens, a continuous photoperiod, or UV radiation (Dietrich *et al.*, 1997; Mateo *et al.*, 2004; Wituszyńska *et al.*, 2015). PCD in *lsd1* depends on ENHANCED DISEASE SUSCEPTIBILITY1 (EDS1), since null mutation in *EDS1* reverts to the *lsd1*-conditioned cell death (Rustérucci *et al.*, 2001; Mateo *et al.*, 2004; Mühlenbock *et al.*, 2007, 2008; Wituszyńska *et al.*, 2015). Moreover, the *eds1* mutant has been described to be less sensitive towards UV-C radiation (Wituszyńska *et al.*, 2015).

Rapid climate changes hinder the prediction of perturbations in numerous environmental factors and their implications on ecosystems. More recent studies have focused on searching for mechanisms of UV perception in plants, since UV radiation is one of the most serious and frequent factors inducing plant PCD (Nawkar *et al.*, 2013). UV is conventionally divided into three wavebands: UV-C (200–280nm), UV-B (280–315nm), and UV-A (315–400nm). In fact, this division is somewhat arbitrary, but it is a useful contraction for considering the biological and ecological effects of different UV radiation spectra. As photoautotrophs, plants are inevitably exposed to the damaging effects of UV. It is especially harmful to photosystem II (PSII) and the CO_2_ assimilation process, even more than an excess of white light (Ohnishi *et al.*, 2005). UV-C is strongly absorbed by the oxygen and ozone in the atmosphere, and in theory it should not reach the Earth’s surface. However, in the early history of the Earth, an intense solar UV-C had been a major factor limiting the evolution of early life (Paul and Gwynn-Jones, 2003; Stratmann, 2003). Moreover, UV-C-triggered changes in plant cells are comparable with those induced by UV-B radiation, which reaches Earth’s biosphere (Danon and Gallois, 1998). It has been demonstrated that mitochondria and ROS play an important role in the regulation of the plant cell death programme, induced by UV-C (Gao *et al.*, 2008). Studies on suspension cultures of carrot cells after UV-C exposure have determined three types of cell death: necrosis, apoptotic-like PCD, and autophagy (Balestrazzi *et al.*, 2010), suggesting that the UV-C triggered responses are highly complex, and the interaction of different UV-induced pathways is still poorly understood.

The role of phytochromes in perceiving UV light is not well recognized, but it has been shown that phyA can mediate UV-A-dependent chloroplast gene transcription (Chun *et al.*, 2001). Therefore, the role of phytochromes in the regulation of downstream responses to other UV spectra (UV-B and UV-C) is plausible. In this study, we formulated a hypothesis that phyA and phyB play a role in the regulation of UV-C-triggered PCD. To the best of our knowledge, this is the first study that presents such a broad characterization of phyA- and phyB-deficient mutants, focusing on photosynthetic efficiency and ROS/SA homeostasis under UV-C-induced cell death.

This study is divided into three parts. The first shows differences in photosynthesis parameters in phytochrome mutants in comparison with the wild-type plants. The second describes how phytochrome mutants react to UV-C radiation stress in terms photosynthesis and antioxidant system deregulation, and ultimately cell death. The last part shows some insight into transcriptional regulation and presents common genes involved in phyA- and phyB-mediated pathways and plant responses to UV stress.

## Materials and methods

### Plant material and growth conditions

*Arabidopsis thaliana* wild-type (ecotype Col-0) and three lines of phytochrome null mutants, all in the Col-0 background, were used. *phyA-211*, *phyB-9*, and double mutant *phyA-211 phyB-9* previously described by Reed *et al.* (1993, 1994) were kindly provided by Professor E. Schäfer (Institut für Biologie II, Universität Freiburg, Germany). Additionally, *lsd1* (*lesion simulating disease 1*) and *eds1* (*enhanced disease susceptibility 1*) mutants in the Col-0 background were used as a positive and negative control of PCD, respectively, as described previously (Wituszyńska *et al.* 2015). After 3 d of stratification at 4 °C, seeds were grown on Jiffy Peat Pellets (Jiffy Products International, The Netherlands) under the following conditions: 8/16h photoperiod, 22/18 °C (day/night), relative air humidity of 70±5%, and light intensity 90±5μmol m^–2^ s^–1^. In all experiments, 3- to 4-week-old plants were used.

### UV treatment

A UV-C 500 Crosslinker (Hoefer Pharmacia Biotech, USA), equipped with a Sankyo Denki lamp (type G8T5, 8W each; Japan), was used for plant UV exposure. The UV spectrum ranged from 250 to 258nm, with the maximum at 253.7nm. Plants were exposed to UV at a dose of 100 or 200 mJ cm^–2^. Dry weight was determined for 4-week-old plants. Whole rosettes were collected, dried at 105 °C for 3h, and each rosette was weighed.

### Gas exchange and chlorophyll *a* fluorescence parameter measurements

Gas exchange and chlorophyll *a* fluorescence parameters were measured simultaneously on whole rosettes using a Portable Gas Exchange Fluorescence Systems GFS-3000 (Heinz Walz GmgH, Germany). Gas exchange parameters were measured at variable light intensity (20–1700 μmol m^–2^ s^–1^) and intercellular CO_2_ concentration (ci) (60–1350 ppm) as described previously (Wituszyńska *et al.*, 2013a). Before the first level of photosynthetically active radiation (PAR) was applied, each plant was dark adapted for 30min. During modulated light measurements, the CO_2_ concentration in the plant cuvette was maintained at 380 ppm. In modulated ci, PAR was maintained at 600 µmol m^–2^ s^–1^. Variables such as PAR, air temperature, leaf temperature, and vapour pressure deficit were recorded together with fluorescence intensity (*F*_o_, *F*_m_, *F*_s_, *F*_m_’) and were used for calculations of gas exchange and fluorescence parameters. Following Caemmerer and Farquhar (1981), variations in gas exchange and fluorescence parameters were presented depending on PAR or ci. For regression analysis of PAR and ci curves, the following measuring points were used: 20, 100, 300, 600, 900, 1200, and 1700 µmol m^–2^ s^–1^, and 60, 100, 165, 320, 650, and 1350 ppm, respectively. At each PAR or ci, readings of the following parameters were collected or calculated according to the manufacturer’s instructions: *A*, assimilation rate; ci, the intercellular molar fraction of CO_2_; GH_2_O, water vapour conductance; ΦPSII, quantum yield efficiency of photosystem II; and non-photochemical quenching (fluorescence quenched by other processes than photochemistry) measured as qN (the non-photochemical fluorescence quenching determined in relation to the maximal variable fluorescence; Schreiber *et al.*, 1986) or NPQ (the non-photochemical fluorescence quenching determined in relation to the remaining maximal fluorescence; Bilger and Björkman, 1990). The results of assimilation rate and stomatal conductance were calculated per unit leaf area measured with a FlourCam system (PSI, Czech Republic). Statistical analysis was performed in R, version 2.12.1 using the statistics packages. To compare the regression line slopes, the procedure from Wonnacott and Wonnacott (1990) based on the use of a mute variable *D* was used.

### Stomatal density

Imprints of the abaxial leaf side were taken using transparent glue (UHU, Germany). For each genotype, three leaves (leaves 6, 7, and 8) from three different plants were analysed. Images were taken with a confocal microscope (LSM-700; Zeiss, Germany), using the light pathway. The number of stomata mm^–2^ of leaf area was calculated from three frames of each microscopic sample.

### Electrolyte leakage measurement

Cell death was quantified by cellular electrolyte leakage from the whole rosettes. The assay was conducted according to Overmyer *et al.* (2000). Three-week-old plants were transferred to 50ml Falcon tubes filled with 35ml of deionized water. The conductivity of the solution (μS cm^–1^) in relation to the rosette fresh weight (g) was determined after 1h using a conductivity meter (InoLab Cond Level 1; WTW, Germany).

### Trypan blue staining

Trypan blue staining was used to detect the level of plant cell death *in situ*. For each genotype, three leaves (leaves 6, 7, and 8), from three different plants were analysed. Staining was performed for non-treated and UV-treated plants, according to Idänheimo *et al.* (2014) with minor modifications. Freshly detached leaves were boiled with trypan blue/lactophenol solution for 3min and subsequently washed in 15M chloral hydrate solution. Samples were stored in 60% glycerol and examined by stereomicroscopy.

### Hydrogen peroxide and superoxide levels determination

Hydrogen peroxide (H2O2) level was determined according to Velikova *et al.* (2000) with minor modifications. Fresh tissue (50mg) was homogenized in TissueLyser LT (Qiagen, The Netherlands) (5min, 50 Hz, 4 °C) with 300 µl of cold 0.1% trichloroacetic acid and centrifuged at 13 000rpm for 15min. The supernatant was mixed with 10mM potassium phosphate buffer (pH 7.0) and 1M KI at a ratio of 1:1:2 (v/v/v). Absorbance was measured at 390nm using microplate reader Multiscan-GO (Thermo Scientific, USA) and the concentration of H_2_O_2_ was calculated using an appropriate standard curve. Data were expressed as µmol of H_2_O_2_ per 100mg of dry weight. The visualization of H_2_O_2_ in *Arabidopsis* leaves was performed by infiltration with 5mM 3,3’-diaminobenzidine (DAB) as described previously (Wituszyńska *et al.*, 2015). Nitro blue tetrazolium (NBT) staining was used to detect the production of superoxide radicals (O_2_^.^ˉ). For each genotype, three leaves (leaves 6, 7, and 8), from three differ ent plants were analysed. Staining was performed for non-treated and UV-treated plants, according to Wohlgemuth *et al.* (2002) with minor modifications. Freshly detached leaves were immediately immersed in 50mM potassium phosphate buffer (pH 7.8) containing 0.1% NBT and 10mM sodium azide. The leaves were vacuum infiltrated for 2min and incubated for 2h in the dark (without vacuum). After incubation, they were immersed in glycerol:acetic acid:ethanol mixture (1:1:3, v/v/v) to completely eliminate chlorophyll. Superoxide production was visualized as a dark blue formazan deposit within the leaf tissue.

### Protein extraction

Frozen leaf tissue (50–100mg) was homogenized in TissueLyser LT (Qiagen) (5min, 50 Hz, 4 °C) with 1ml of extraction buffer (100mM Tricine, 3mM MgSO_4_, 3mM EGTA, 1mM dithiothreitol, 1M Tris/HCl, pH 7.5). Samples were incubated on ice for 15min and centrifuged at 13 000rpm for 20min. The enzyme extract for the APX activity assay additionally contained 5mM l-ascorbic acid. Protein concentration was determined using a Bradford assay kit (Thermo Scientific) with BSA as a standard.

### Enzymes activity measurements

Spectrophotometric assay of superoxide dismutase (SOD) activity was performed according to Beauchamp and Fridovich (1971) with modifications. Enzyme assay mixture contained 0.1M phosphate buffer (pH 7.5), 2.4 µM riboflavin, 840 µM NBT, 150mM methionine and 12mM Na_2_EDTA at a ratio of 8:1:1:1:1 (v/v/v/v/v). The enzyme extract was mixed with the enzyme assay mixture in such a proportion that the inhibition of the NBT oxidation was in the range of 20–80%. Absorbance was measured at 560nm, 15min after sample illumination with 500 µmol m^–2^s^–1^ (LED Lamp SL3500-W-D; PSI) or incubation in the darkness (blank sample). Results were expressed in units (amount of enzyme that inhibited NBT photoreduction to blue formazan by 50%) (mg of protein)^–1^. The activity of catalase (CAT) was measured spectrophotometrically according to Aebi (1984) with modifications. Perhydrol was diluted with 50mM phosphate buffer (pH 7.0) to the absorbance of 0.5 (±0.02) at 240nm (initial concentration of H_2_O_2_ corresponding to about 13mM). The enzyme extract was mixed with 50mM phosphate buffer in such a proportion that absorbance decreased to the range of 20–80%. CAT activity was measured as the rate of H_2_O_2_ decay within 2min and calculated using the molecular extinction coefficient of H_2_O_2_ at 240nm (ε=43.6mol^–1^ cm^–1^). Results were expressed in µmol of H_2_O_2_ min^–1^ (mg of protein)^–1^. The activity of ascorbate peroxidase (APX) was measured spectrophotometrically according to Nakano and Asada (1981) with modifications. The enzyme extract was mixed with the assay buffer (50mM phosphate buffer, pH 7.0, 1mM EDTA, 15mM l-ascorbic acid, 40mM H_2_O_2_) in such a proportion that absorbance decreased to the range of 20–80%. APX activity was measured as the rate of ascorbate decay within 2min and calculated using the molecular extinction coefficient of ascorbate at 290nm (ε=2.8 mmol^–1^ cm^–1^). Results were expressed in µmol of l-ascorbic acid min^–1^ (mg of protein)^–1^. Spectrophotometric measurements were performed using a UV-VIS microplate reader Multiscan-GO (Thermo Scientific).

### Salicylic acid determination

The determination of salicylic acid (SA) was performed as described by Meuwly and Métraux (1993). 2-Methoxybenzoic acid and 3-hydroxybenzoic acid were used as the internal standards. SA was eluted on a Luna 5 μm C18(2) 100Å 150×4.6mm column (Phenomenex, USA) at 30 °C for 15min using a Shimadzu HPLC System (Shimadzu, Japan). Low-pressure gradient system was used with 20mM phosphate buffer (pH 2.5; adjusted with 8M HCl) and acetonitryle (75:25; v/v) at a flow rate of 1ml min^–1^. Results were expressed as µg of SA (g of dry weight)^–1^.

### Chlorophyll *a* fluorescence measurements

The measurement of chlorophyll *a* fluorescence was performed on whole *Arabidopsis* rosettes using the FluorCam system (PSI, Czech Republic). For both, quenching and OJIP tests, dark-adapted plants (30min) were used to determine *Fv/Fm*, qN, and NPQ. However, during the chlorophyll fluorescence measurement protocol, the light-adaptation phase was also included, which enabled to determine other light-dependent parameters such as ΦPSII. Chlorophyll *a* fluorescence parameters have been described in details elsewhere (Baker, 2008; Wituszyńska *et al.*, 2013a).

### Photosynthetic pigments measurements

Frozen tissue (50–100mg) was homogenized in the TissueLyser LT (Qiagen) (5min, 50 Hz, 4 °C) with 1ml of cold acetone (–20 °C). Homogenate was evaporated under N_2_, dissolved in cold solvent A (acetonitrile:methanol; 90:10; v/v), and rehomogenized for 1min. The extract was filtered (0.2 µm nylon filter; GE Healthcare, UK) and stored in the dark at 80 °C until high-performance liquid chromatography analysis (Shimadzu). Pigments were separated on a Synergi 4 μmMAX-RP 80Å 250×4.6mm column (Phenomenex) at 30 °C. Low-pressure gradient system was used: solvent A for 10min to elute all xanthophylls, followed by solvent B (methanol:ethyl acetate; 68:32; v/v) for 10min to elute the rest of pigments. The flow rate was 1ml min^–1^. Absorbance spectra were recorded at 440nm. Pigments were identified using standards (Sigma, Hoffmann-LaRoche, Fluka). Results were expressed as peak area (mg of dry weight)^–1^. The De-epoxidation state was measured as (antheraxanthin+zeaxanthin)/(violaxanthin+antheraxanthin+zeaxanthin).

### Microarray meta-analysis

For the microarray meta-analysis, the data sets of transcripts significantly deregulated (*P*<0.05) in Col-*phyA* and Col-*phyB* mutants in comparison to the wild type (Col-0) were obtained from publicly available microarray results (Mazzella *et al.*, 2005). Data were compared with the NASCARRAYS-124 microarray experiment for UV-C induced transcriptional changes (*P*<0.05) in Col-0 available at the NASC International Affymetrix Service. Transcriptomic data sets were functionally classified using the MapMan 3.5.0 Beta Tool (Thimm *et al.*, 2004) in the search for various cellular pathways.

### Statistics

If not described differently, all results were presented as means±SD of 9–12 plants per genotype from two independent experiments (*n*=18–24). Statistical analysis was performed in STATGRAPHICS Plus 5.1 or in R version 2.13. A Shapiro–Wilk normality test and Bartlett test of variances homogeneity were performed. For parametric tests, analysis of variance was used, while for non-parametric tests, a Kruskal–Wallis test was performed.

## Results

### phyA and phyB are positive regulators of photosynthetic activity

**Fig. 1.**
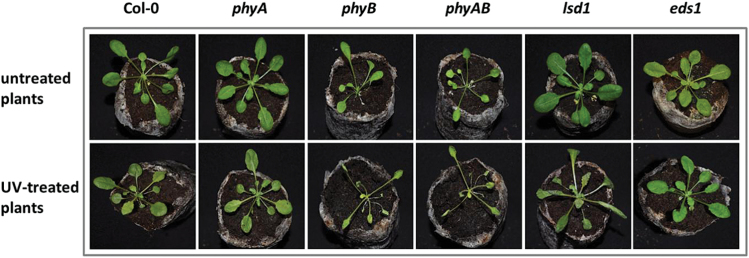
Phenotypes of *A. thaliana* wild-type (Col-0), phytochrome mutants (*phyA*, *phyB*, and *phyAB*), *lsd1*, and *eds1* plants in the Col-0 background. Upper panel, morphology of untreated plants; bottom panel, UV-treated plants, 96h after exposure to 100 mJ cm^–2^ of UV-C.

To investigate the role of phyA and phyB in the photosynthetic regulation, we used simultaneous gas exchange and chlorophyll *a* fluorescence measurements for the wild-type and phytochrome mutants (*phyA*, *phyB*, and *phyAB*). Since *phyB* and *phyAB Arabidopsis* mutants display a constitutive shade-avoiding phenotype characterized by elongated petioles (Reed *et al.*, 1993) (Fig. 1), all the measured parameters were recalculated taking into consideration the rosette area.

**Fig. 2.**
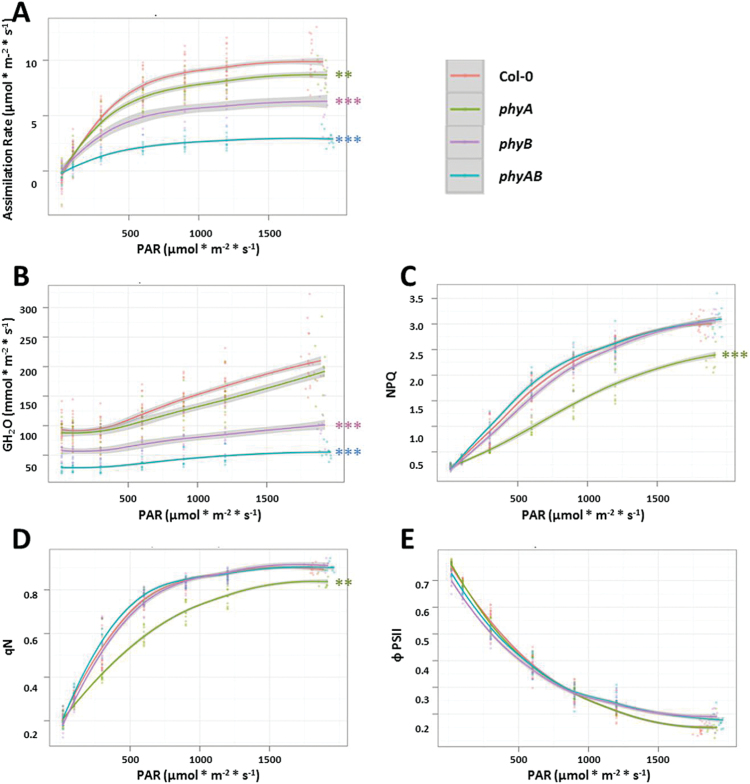
Gas exchange and chlorophyll *a* fluorescence parameters in response to different light intensities (PAR) were measured for wild type and phytochrome mutants. Graphs represent relationships between light intensity and individual photosynthetic parameters. (A) Assimilation rate of CO_2_; (B) stomatal conductance; (C, D) non-photochemical quenching; (E) PSII operating efficiency. Values are means (with 95% confidence intervals marked in grey shading) of 9–12 plants per genotype from two independent experiments (*n*=18–24). Asterisks indicate significant differences of regression line slopes in comparison with the wild type, according to the Tukey HSD test at the level of *P*<0.05 (*), *P*<0.005 (**), or *P*<0.001 (***).

**Fig. 3.**
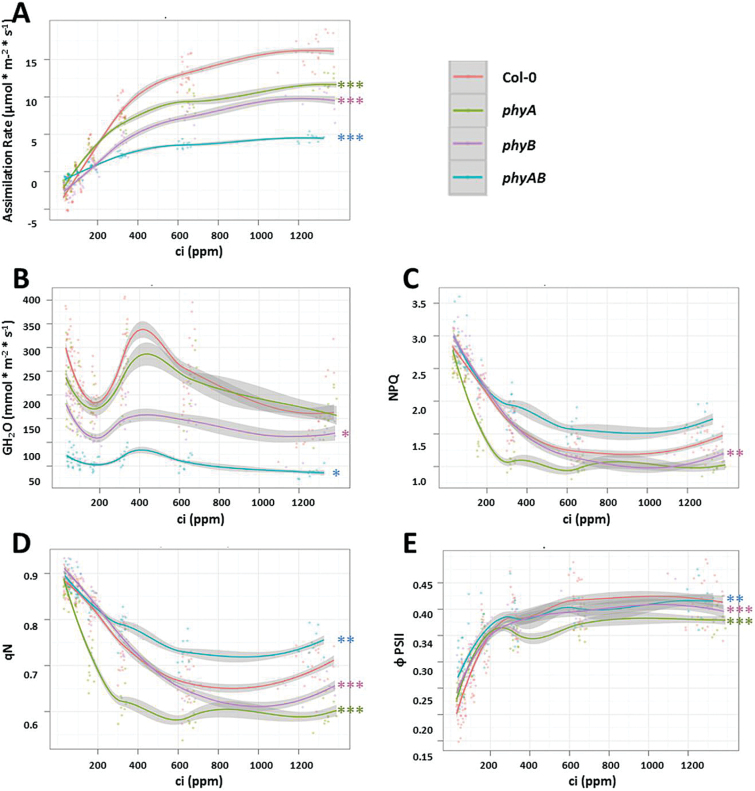
Gas exchange and chlorophyll *a* fluorescence parameters in response to different CO_2_ concentrations (ci) were measured for wild type and phytochrome mutants. Graphs represent relationships between intercellular CO_2_ mole fraction (ci) and individual photosynthetic parameters. (A) Assimilation rate of CO_2_; (B) stomatal conductance; (C, D) non-photochemical quenching; (E) PSII operating efficiency. Values are means (with 95% confidence intervals marked in grey shading) of 9–12 plants per genotype from two independent experiments (*n*=18–24). Asterisks indicate significant differences of regression line slopes in comparison with the wild-type, according to the Tukey HSD test at the level of *P*<0.05 (*), *P*<0.005 (**), or *P*<0.001 (***).

Photosynthetic parameters are presented as the function of light intensity (PAR) or intercellular CO_2_ mole fraction (ci) (Figs 2 and 3, Supplementary Table S2, available at *JXB* online). The *phyA*, *phyB*, and double *phyAB* mutants showed decreased CO_2_ assimilation rate in comparison with Col-0 (Figs 2A and 3A). These differences were most pronounced in *phyAB* (Figs 2A and 3A). Stomatal water vapour conductance (GH_2_O) in changing PAR and CO_2_ concentration was also significantly decreased in *phyB* and *phyAB*, but not in *phyA* (Figs 2B and 3B). Assuming that CO_2_ diffuses from air through the substomatal cavity using the same pathway in which the water vapour escapes, leaf conductance for CO_2_ can be concluded from GH_2_O. Due to the fact that the reduction in assimilation rates in *phyA*, *phyB*, and *phyAB* might be associated with lower leaf GH_2_O in these mutants, we measured stomatal density in all tested genotypes (Supplementary Table S1, available at *JXB* online). We found significantly lower stomatal density in *phyB* and *phyAB*, which can, at least partially, explain the decreased assimilation capacity in these plants. Chlorophyll *a* fluorescence parameters were also changed in phytochrome mutants compared with the wild type (Figs 2C–E and 3C–E). Non-photochemical quenching parameters (NPQ and qN) that represent the excess energy dissipation by heat emission were decreased in *phyA* mutant in growing PAR. The same parameters measured in increasing intercellular CO_2_ were affected in *phy* mutants in comparison with the wild type. Significantly lower NPQ was observed in *phyB*. The qN parameter was decreased in *phyA* and *phyB* but increased in *phyAB*. Non-photochemical quenching reactions are known to diminish the efficiency of photochemistry (Graßes *et al.*, 2002) and are major component of photoprotection (Baker, 2008). PSII operating quantum yield (ΦPSII) provides an estimate of the quantum yield efficiency of PSII in the light (Baker, 2008). In response to increasing light intensity, we did not observe any statistically important differences in ΦPSII among genotypes (Fig. 2E). On the other hand, under modulated CO_2_ concentration, all *phy* mutants showed decreased ΦPSII (Fig. 3E).

The above data indicated that phyB and phyA plus phyB, but not phyA alone positively regulate stomata development (Supplementary Table S1) and thus affect CO_2_ assimilation and stomatal conductance. Moreover, phyA and phyB influence non-photochemical reactions and PSII efficiency, but this regulation seems to be complex and condition dependent. Our results showed the genetic relationship between phyA and phyB, and their role in the mechanisms controlling photosynthesis efficiency. Since differences in photosynthetic capacity are frequently associated with altered sensitivity to environmental stresses, we tested the response of *phy* mutants towards oxidative stress caused by UV-C radiation.

### phyA and phyB regulate responses to UV-induced stress

In order to study the role of phyA and phyB in the induction of cell death triggered by UV-C radiation, electrolyte leakage measurement of UV-treated versus untreated plants was performed. Two different UV doses, i.e. 100 and 200 mJ cm^–2^, were used and the physiological effects of UV radiation were observed before and after UV-C stress.

**Fig. 4.**
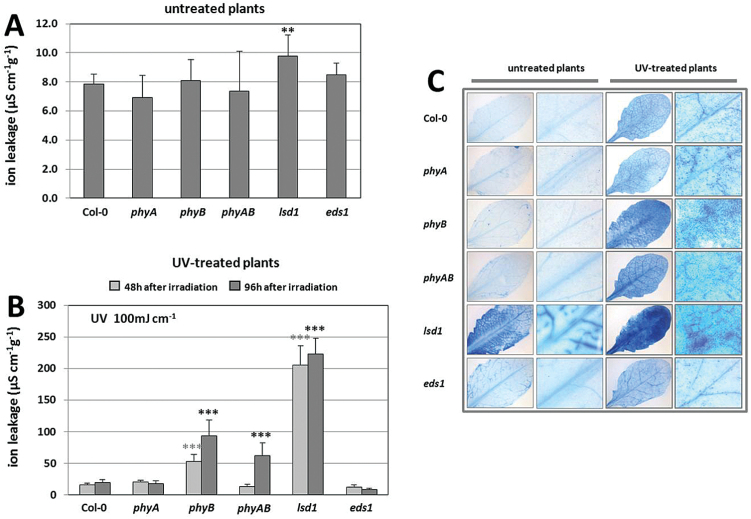
(A, B) Cell death expressed as ion leakage was determined for untreated plants (A) and UV-treated plants (B), 48 and 96h after exposure to 100 mJ cm^–2^ of UV. Values are means±SD of 9–12 plants per genotype from two independent experiments (*n*=18–24). Asterisks indicate significant differences from the wild type according to the Tukey HSD test at the level of *P*<0.05 (*), *P*<0.005 (**) and *P*<0.001 (***). (C) Trypan blue staining as an indicator of cell death was visualized 96h after UV treatment.

Untreated control plants demonstrated no differences in ion leakage, with the exception of *lsd1* mutant (Fig. 4A). *lsd1* and *eds1* mutants were used as a positive and negative control of UV-triggered cell death, respectively (Wituszyńska *et al.*, 2015). UV-C radiation significantly increased the ion leakage in *lsd1*, *phyB* and *phyAB* (Fig. 4B and Supplementary Fig. S1, available at *JXB* online). The highest ion leakage for these mutants was observed 96h after the UV-C episode, in both UV-C doses. We found that *phyB* was most sensitive to UV-C, as it showed about 3-fold higher ion leakage 48h after radiation with 100 mJ cm^–2^ of UV-C and about 5-fold after 96h (Fig. 4B). Increasing the radiation dose to 200 mJ cm^–2^ further enhanced ion leakage, but the statistically significant differences between *phyB* and Col-0 were observed only 96h after UV exposure and surprisingly were less pronounced (about 1.5-fold) (Supplementary Fig. S1). These findings were confirmed by the visualization of dead cells using trypan blue staining. Our results demonstrated that, after UV treatment, most dark blue spots, which are the signs of dead cells, were present in *lsd1* mutant. *phyB* and *phyAB* mutants also showed the high level of dead cells, which was consistent with the electrolyte leakage results. The lowest level of cell death was observed for *phyA* and *eds1* mutants. Since the phenotypes of *lsd1* and *phyB* are both characterized by accelerated induction of cell death, it was concluded that LSD1 and phyB can act as negative regulators of PCD. There were no differences in the ion leakage between Col-0 and *phyA* (Fig. 4 and Supplementary Fig. S1). Furthermore, lower level of electrolytic leakage in the double *phyAB* mutant, compared with *phyB*, may indicate the positive role of phyA in cell death regulation and an antagonistic regulatory role of phyA and phyB in this process.

**Fig. 5.**
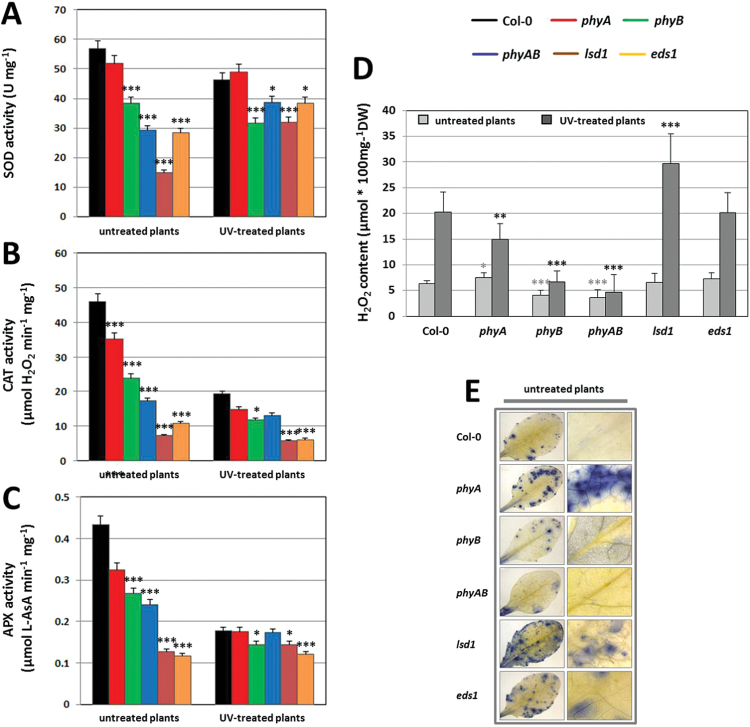
(A–C) Activities of selected antioxidant enzymes: Superoxide dismutase (SOD) (A), catalase (CAT) (B), and ascorbate peroxidase (APX) (C) determined for untreated plants and 96h after UV-C exposure (100 mJ cm^–2^). Values are means±SEM of 9–12 plants per genotype from two independent experiments (*n*=18–24). Asterisks indicate significant differences in comparison with Col-0 according to the Tukey HSD test at the level *P*<0.05 (*), *P*<0.005 (**), and *P*<0.001 (***). (D) H_2_O_2_ concentration in untreated and UV-treated plants, 96h after UV-C exposure (100 mJ cm^–2^). (E) Superoxide visualization in untreated plants by NBT staining.

In all further experiments, we used the UV dose of 100 mJ cm^–2^ and measurements were performed before and 96h after UV-C exposure. Apart from phytochrome mutants, *lsd1* and *eds1* were also subjected to the analysis as they demonstrate sensitivity or resistance towards UV-induced cell death, respectively (Wituszyńska *et al.*, 2015). Qualitative and quantitative determination of foliar ROS was performed for untreated and UV-treated plants. We found that O_2_^.^ˉ and H_2_O_2_ content differed in *phy* mutants even before UV-C stress (Fig. 5D, E, and Supplementary Fig. S2, available at *JXB* online). Non-treated *phyB* and *phyAB* exhibited 35 and 43% lower H_2_O_2_ content compared with control plants, respectively (Fig. 5D). This result was confirmed by DAB staining (Supplementary Fig. S2). The O_2_^.^ˉ content in these mutants was also lower (Fig. 5E). In contrast, the H_2_O_2_ concentration in *phyA* was 19% higher than in the wild type (Fig. 5D), which was confirmed by DAB staining (Supplementary Fig. S2). O_2_^.^ˉ in *phyA* demonstrated the same pattern (Fig. 5E). There was no significant difference in H_2_O_2_ content between *lsd1* and *eds1* in comparison to Col-0 before UV stress (Fig. 5D). H_2_O_2_ concentration 96h after UV stress was significantly lower in all *phy* mutants. Compared with the wild-type plants, it was 26% decreased in *phyA* and 67% lower in *phyB* and even more in *phyAB* (Fig. 5D). These results were in agreement with the H_2_O_2_ visualization by DAB staining (Supplementary Fig. S2). 96h after UV-C treatment, there was only little superoxide in the leaf tissue (Supplementary Fig. S3 available at *JXB* online), most probably because of relatively short half-life of O_2_^.^ˉ (op den Camp *et al.*, 2003) and advanced PCD expansion.

The activities of main antioxidant enzymes that keep cellular ROS homeostasis under tight control were evaluated before (0h) and 96h after UV-C treatment (Fig. 5A–C). We found that the deficiency of phytochromes affected the foliar levels of antioxidant enzymes both, before and after UV-C stress.

SOD activity represents the first step in the ROS scavenging pathway, detoxifying O_2_^.^ˉ into H_2_O_2_ and O_2_ (Tsang *et al.*, 1991; Alscher *et al.*, 2002). Before UV stress, all analysed mutants demonstrated reduced SOD activity in comparison with Col-0 (Fig. 5A). The lowest SOD activity was observed in *lsd1* plants (decreased 3.8-fold). *eds1* and *phyAB* showed comparable SOD activities, both about 50% lower than Col-0. In *phyB*, the enzyme activity was 32% reduced, while in *phyA* only a slight drop was found. Significant reduction in SOD activity measured 96h after UV-C treatment was shown for *phyB* and *lsd1* when compared with Col-0 (31 and 32% lower, respectively). Smaller decrease, about 16–17%, was shown in *eds1* and *phyAB*. *phyA* did not demonstrate statistically significant differences (Fig. 5A).

In non-treated plants, CAT and SOD activities correlated within a particular genotype. In all mutants, CAT activity was reduced compared with Col-0 before UV-C stress (Fig. 5B). 96h after UV-C, the highest decrease in CAT activity was observed in *lsd1* and *eds1*, whereas the other mutants (*phyA*, *phyB* and *phyAB*) had 22–39% lower CAT activity (Fig. 5B). However, only in *phyB* mutant this difference was significant.

Similarly, the APX activity correlated with the results for CAT and SOD in non-treated plants. Before UV-C stress, all mutants demonstrated a decrease in APX activity compared with Col-0. However, in *phyA* this reduction was not significant. The highest decrease was found in *lsd1* and *eds1* (70–73%) (Fig. 5C). Among phytochrome mutants, the lowest APX activity in comparison with the wild type was exhibited by *phyB* and *phyAB* (around 38–44% decrease). The differences at 96h after UV-C were less clearly pronounced. APX activity in *phyA* and *phyAB* was comparable to that in Col-0, whereas *phyB*, *lsd1*, and *eds1* showed significantly lower APX activity (Fig. 5C).

**Fig. 6.**
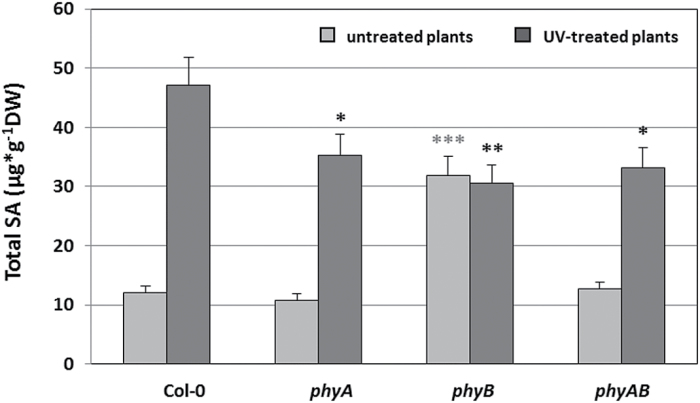
Salicylic acid content measured in untreated and UV-treated plants, 96h after UV-C radiation (100 mJ cm^–2^). Values are means±SD of 9–12 plants per genotype from two independent experiments (*n*=18–24). Asterisks indicate significant differences from the wild type according to the Tukey HSD test at the level of *P*<0.05 (*), *P*<0.005 (**), or *P*<0.001 (***).

The significance of interaction between SA, H_2_O_2_, and H_2_O_2_-metabolizing enzymes with oxidative damage and cell death has been presented in many studies (Mateo *et al.*, 2006; Rivas-San Vicente and Plasencia, 2011; Petrov and Van Breusegen, 2012). Since the differences in H_2_O_2_ content and antioxidant enzymes activities were dependent on phytochromes, we also compared SA concentrations in mutants and wild-type plants. Under standard conditions, untreated *phyB* demonstrated 2.7-fold higher SA content compared with the wild type. This effect was reversed by the mutation in *PHYA*, since both *phyA* and *phyAB* double mutants had SA levels comparable to the wild-type plants (Fig. 6). These results suggests that phyA may act antagonistically to phyB in SA synthesis/regulation in non-stress conditions. After UV-C stress, significantly lower SA concentrations were observed for all *phy* mutants in comparison with Col-0 (Fig. 6). This observation may indicate the role of phytochromes in SA accumulation/signalling during stress conditions. Altogether, our results showed an important role of phyA and phyB in SA cellular homeostasis maintenance.

### UV-C triggered changes in photosynthetic apparatus are dependent on phyA and phyB

Imaging methods for chlorophyll *a* fluorescence provide a simple tool for *in vivo* monitoring of structural and functional state of photosynthetic apparatus, from which plant physiological status can be assumed (Gawroński *et al.*, 2013; Wituszyńska *et al.*, 2013a). To estimate the level of plant damage caused by UV-C radiation, we performed analyses of chlorophyll *a* fluorescence parameters, before and 96h after plants ‘exposure to UV-C (100 mJ cm^–2^).

**Fig. 7.**
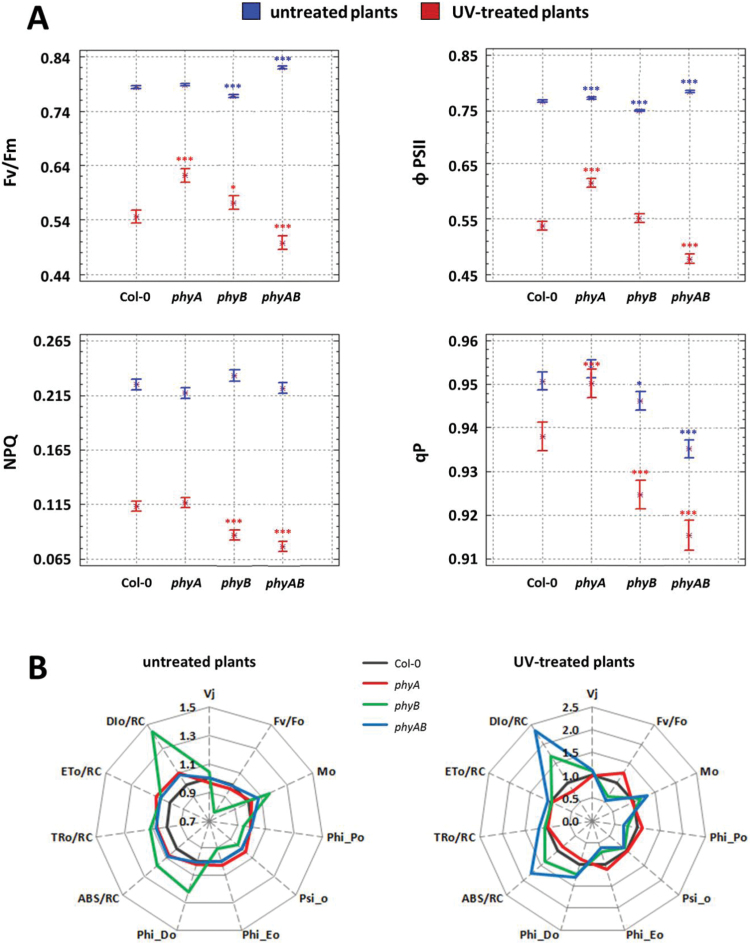
Chlorophyll *a* fluorescence parameters measured for untreated and UV-C treated plants (96h after 100 mJ cm^–2^ UV-C dose) in wild-type and phytochrome mutants. (A) Fluorescence parameters calculated from chlorophyll *a* fluorescence induction curve. (B) Fluorescence parameters from the OJIP test. Values are means±SD of 9–12 plants per genotype from two independent experiments (*n*=18–24). Asterisks in (A) indicate significant differences from the wild type according to the Tukey HSD test at the level of *P*<0.05 (*), *P*<0.005 (**), or *P*<0.001 (***). For full statistical analysis results of (B) see Supplementary Table S2. *F*_v_/*F*_m_, maximum quantum efficiency of PSII; ΦPSII, quantum yield of PSII; NPQ, non-photochemical quenching; qP, photochemical quenching; *V*_j_, relative variable fluorescence at the J-step; *F*_v_/*F*_o_, efficiency of the oxygen-evolving complex; *M*_o_, approximated initial slope of the fluorescence transient; Phi_P_0_, maximum quantum yield of primary photochemistry; Psi__0_, probability that a trapped exciton moves an electron into the electron transport chain beyond Q_A_^–^; Phi_E_0_, quantum yield of electron transport; Phi_D_0_, quantum yield of energy dissipation; ABS/RC, absorption flux per reaction centre; TR_0_/RC, trapped energy flux per reaction centre; ET_0_/RC, electron transport flux per reaction centre; DI_0_/RC, dissipated energy flux per reaction centre.

Untreated *phyB* plants had lower value of the maximum quantum efficiency of PSII (*F*_v_/*F*_m_), quantum yield of PSII (ΦPSII), and photochemical quenching (qP) in comparison with Col-0 (Fig. 7A). *phyAB* mutant demonstrated increased *F*_v_/*F*_m_ and ΦPSII, but surprisingly its capacity for photochemical quenching was strongly decreased in comparison with the wild-type plants (Fig. 7A). *phyA* differed significantly from the wild type only in the higher ΦPSII value. Before stress, there was no difference in the rate of excess energy dissipation through non-photochemical quenching (NPQ) between genotypes (Fig. 7A). After UV-C exposure, *phyA* demonstrated higher values of *F*_v_/*F*_m_, ΦPSII and qP in comparison with the wild-type plants. No significant change in NPQ was observed in this mutant (Fig. 7A). UV-treated *phyB* showed increased *F*_v_/*F*_m_, no alteration in ΦPSII, and decreased qP value. The most severe UV-triggered changes in photosynthetic parameters were demonstrated by *phyAB*. Double mutant showed significantly lower *F*_v_/*F*_m_, ΦPSII, and qP compared with Col-0 (Fig. 7A). Both *phyB* and *phyAB* had also decreased NPQ values after UV-C treatment (Fig. 7A). Taken together, the phytochrome mutants exhibited prominent but variable changes in photosynthetic parameters before and after UV-C stress. These results indicats independent and partially antagonistic involvement of phyA and phyB in photosynthetic electron transport regulation in the proximity of PSII.

A more detailed description of the role of phyA and phyB in the regulation of photosynthetic activity was performed using the OJIP test, a dark-adapted chlorophyll fluorescence technique used for plant stress measurement. All the measured parameters were presented in the radar charts as the ratios of values for individual phytochrome mutants to Col-0 (Fig. 7B). We found significant differences between genotypes in their PSII physiological states both before and after UV-C radiation.

In non-stress conditions, all of the phytochrome mutants demonstrated considerable changes in absorption flux per reaction centre (ABS/RC), trapped energy flux per reaction centre (TRo/RC), and electron transport flux per reaction centre (ETo/RC) in relation to Col-0. However, these functional and structural parameters proved to differ most significantly in *phyB* (Fig. 7B, Supplementary Table S3, available at *JXB* online). The ABS/RC parameter represents the ratio of the total number of photons absorbed by chlorophylls of all RCs in PSII to the number of active RCs. Before UV exposure, increased ABS/RC values were observed in all phytochrome mutants. Additionally, *phyB* and *phyAB* demonstrated higher ABS/RC ratio after UV stress, which indicated decreased number of active reaction centres in these genotypes. Increased TRo/RC suggests that the reduction of plastoquinone Q_A_ pool was stronger in all phytochrome mutants before stress and only in *phyAB* after UV exposure. The ETo/RC ratio estimates the re-oxidation of reduced Q_A_ via electron transport in an active centre of PSII. Because ETo/RC is obtainable only by the active RCs, higher ratios in all phytochrome mutants before stress suggests that they had more inactive centres in comparison with the wild type. After UV-C treatment, ETo/RC for *phyA*, *phyB*, and *phyAB* was comparable to that of Col-0. The DIo/RC parameter characterizes the ratio of the total dissipation of untrapped excitation energy according to the number of active RCs. Strongly increased DIo/RC in *phyA* and *phyB* before UV stress and in *phyB* and *phyAB* after UV treatment indicated that the PSII RCs of these mutants were not efficient in trapping photons. Higher dissipation per active RC can also reflect worse connectivity between the heterogeneous units of PSII (Force *et al.*, 2003). The *V*j parameter, which represents the ratio of closed RCs fraction to the proportion of the total number of RCs, was changed in *phyA* and *phyB* in non-stress conditions. Decreased *V*_j_ in *phyA* suggested that the mutant had proportionally more open centres whereas *phyB* with increased *V*_j_ had less, compared with the wild type. Reduction of the *F*_v_/*F*_o_ ratio indicated that the efficiency of oxygen-evolving complex (OEC) was lower in *phyB* before stress and in both *phyB* and *phyAB* after UV exposure in relation to the wild-type plants. Additionally, before and after UV stress, *phyB* and *phyAB* demonstrated faster accumulation of closed RCs (*M*_o_) than in Col-0. All parameters expressing the quantum efficiencies, the maximum quantum yield of photochemistry (Phi_Po), the quantum yield of electron transport (Phi_Eo), and the probability that a trapped exciton enters the electron transport chain (Psi_o), were lower in *phyB* before UV exposure. The Phi_Po and Psi_o parameters were also significantly lower in *phyB* and *phyAB* after UV treatment. Moreover, *phyA* displayed higher Phi_Po and Psi_o after stress. This can suggest that the inhibition of photosynthetic electron transport observed in *phyB* and *phyAB* after UV stress may have originated from increased UV sensitivity of the light-dependent reactions (represented by Phi_Po) and reactions running behind Q_A_ (represented by Psi_o) (Fig. 7B). Taken together, UV-C treatment resulted in a general decrease in photosynthetic efficiency, mainly in *phyB* and *phyAB*, suggesting their higher sensitivity towards UV-C stress. UV-C-triggered perturbations within the photosynthetic apparatus in these mutants might exceed their PSII repair capacity. To confirm this, further stress/recovery experiments should be performed. Nevertheless, these results point to the importance of phyB in maintaining the structural organization of PSII after stress. In contrast, the *phyA* mutant response to UV-C was less severe than the other genotypes. Moreover, the *phyA* mutation was insufficient to reverse the *phyB* phenotype in *phyAB* double mutant, suggesting a lesser role of phyA in photosynthetic apparatus functional regulation.

**Fig. 8.**
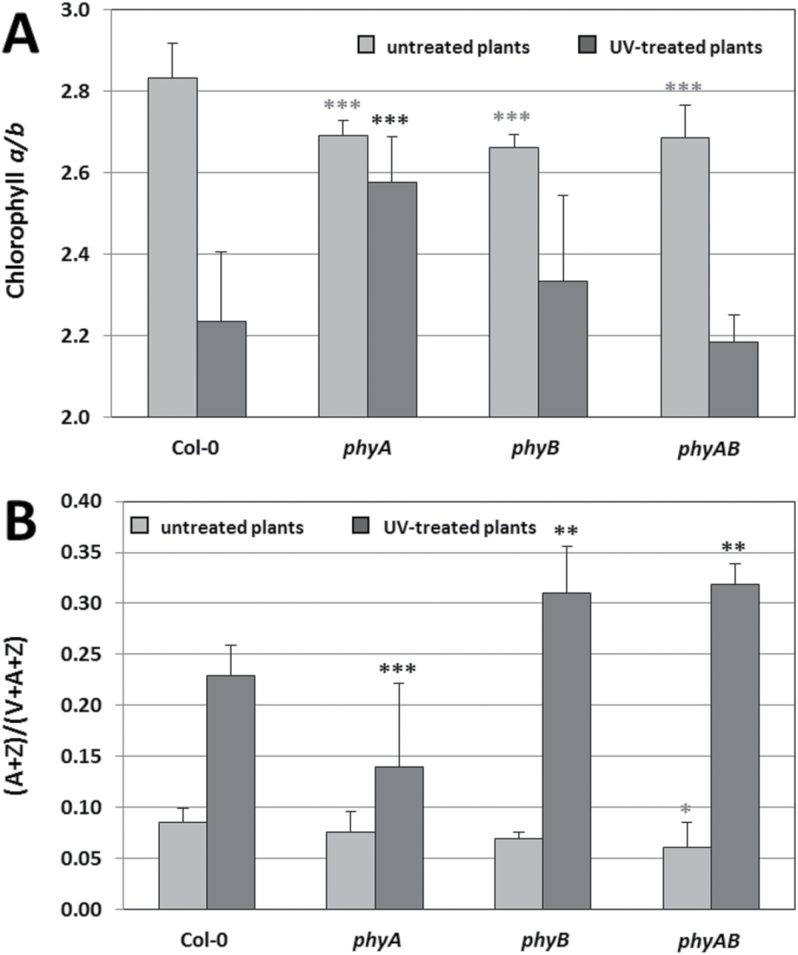
(A, B) Chlorophyll *a/b* ratio (A) and de-epoxidation state (B) of untreated and UV-treated plants (96h after 100 mJ cm^–2^ UV-C dose) in wild-type and phytochrome mutants. Values are means±SD of 9–12 plants per genotype from two independent experiments (*n*=18–24). Asterisks indicate significant differences from the wild type according to the Tukey HSD test at the level of *P*<0.05 (*), *P*<0.005 (**), or *P*<0.001 (***).

Significant changes in fluorescence parameters in phytochrome mutants inspired us to test the composition of their photosynthetic pigments. The average values for chlorophyll and carotenoid content, evaluated before and after UV-C stress, are presented in Table 1. Additionally, the chlorophyll *a*/*b* ratio was calculated (Fig. 8A). The level of chlorophyll *a* and *b* was not significantly changed in *phyA* nor in *phyAB*, under both control and stress conditions. In contrast, *phyB* demonstrated significantly lower chlorophyll content than the wild type, but this effect was observed only before UV-C treatment. These results suggests that phyB positively regulates appropriate chlorophylls concentrations in non-stress conditions. The average chlorophyll *a*/*b* ratio under control conditions was significantly decreased in all phytochrome mutants in comparison with Col-0. In all genotypes, UV-C radiation resulted in a considerable decrease in the chlorophyll *a*/*b* ratio. The highest reduction was observed in the wild-type where the chlorophyll *a*/*b* ratio was ~20% lower than before UV stress. For *phyB* and *phyAB*, a 12.5 and 19% diminished ratio of chlorophyll *a*/*b* was found, respectively, whereas for *phyA* the ratio change was around 5%.

**Table 1.** Chlorophylls and carotenoids contents in un*treated and UV-treated plants* (*96 h* after 100 mJ cm^–2^ UV-C dose) *in wild-type plants and phytochrome mutants* Values are means±SD of 9–12 plants per genotype from two independent experiments (*n*=18–24) expressed as the peak area (mg of dry weight)^–1^. Asterisks indicate significant differences from the wild type according to the Tukey HSD test at the level of *P*<0.05 (*), *P*<0.005 (**), or *P*<0.001 (***).

Pigment	Col-0	*phyA*	*phyB*	*phyAB*
**Untreated plants**				
Chlorophyll *a*	27.5±6.84	22.1±6.99	14.4±1.90***	29.2±5.25
Chlorophyll *b*	9.7±2.16	8.2±2.53	5.4±0.68***	10.9±1.99
β-Carotene	7.7±1.02	5.9±1.71*	6.2±0.60*	8.8±0.76
Lutein	26.1±3.59	25.3±9.65	19.2±2.26	24.0±7.05
Neoxanthin	3.4±0.31	3.2±0.31	2.1±0.13***	3.5±0.33
**UV-treated plants**				
Chlorophyll *a*	19.3±5.10	21.4±8.81	18.6±4.01	19.3±1.23
Chlorophyll *b*	8.8±2.97	8.2±3.16	8.1±2.06	8.8±0.34
β-Carotene	4.5±1.27	4.3±1.49	4.1±0.63	3.4±0.36
Lutein	15.0±5.98	11.9±2.80	11.1±3.55	10.7±1.63
Neoxanthin	2.5±0.79	2.0±0.42	1.8±0.49*	1.5±0.37**

In control conditions, the β-carotene level was significantly lower in both *phyA* and *phyB* in comparison with Col-0 (Table 1). Interestingly, it was not statistically changed in *phyAB*, which suggests a complex phytochrome participation in the regulation of β-carotene content. After UV-C exposure, the β-carotene concentration did not differ among genotypes. The lutein content was not dependent on the genotype nor the stress treatment. Reduction of neoxanthin levels was observed in *phyB* before and after UV-C stress, and additionally in *phyAB* treated with UV-C.

The violaxanthin (V), antheraxanthin (A), and zeaxanthin (Z) concentrations were used to determine the de-epoxidation state (A+Z/V+A+Z) in the tested genotypes. Before UV-C exposure, the average A+Z/V+A+Z ratio was comparable to that of the wild type in *phyA* and *phyB* and was slightly lower in *phyAB*. Nevertheless, 96h after UV-C exposure, *phyB* and *phyAB* showed significantly increased de-epoxidation state, while *phyA*-significantly decreased, in comparison with Col-0 (Fig. 8B). These results indicats that phyB negatively regulates the rate of de-epoxidation, whereas phyA acts positively. Moreover, phyB seems to have more important role on A+Z/V+A+Z since the ratio in the single *phyB* and double *phyAB* mutants was similar.

### phyA and phyB commonly regulate UV-C responsive genes

**Fig. 9.**
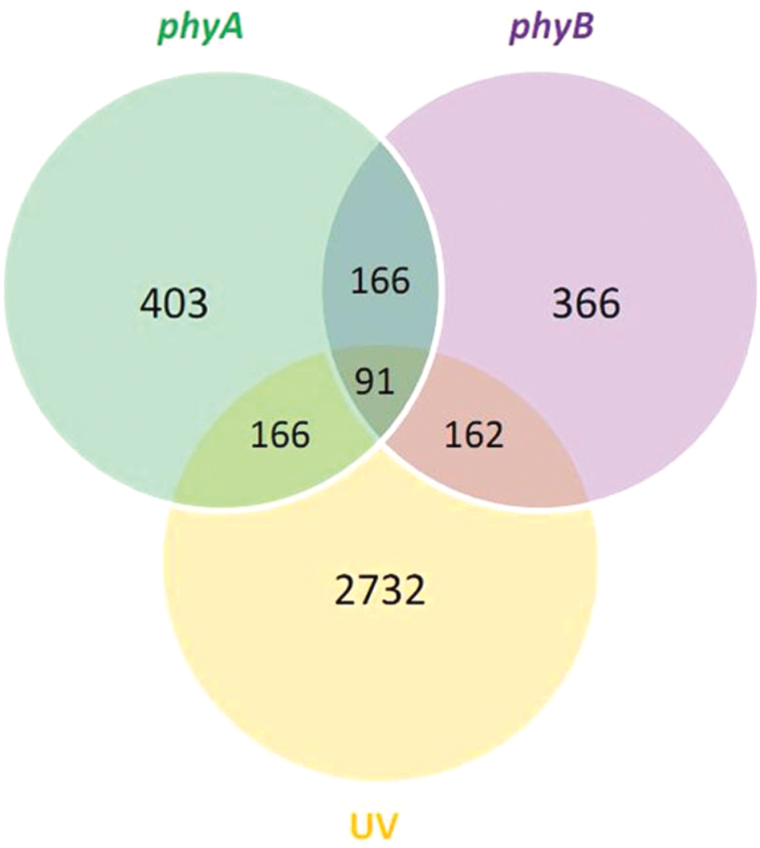
Comparison between transcripts deregulated in *phyA*, *phyB*, and UV-treated wild-type (Col-0) plants. The Venn diagram shows the numbers of commonly regulated genes.

To identify genes that are commonly deregulated in phyA- and phyB-dependent signalling pathways and in the UV-C stress response, we performed a comparison of transcriptomic data available for *Arabidopsis phyA* and *phyB* with expression data for UV-treated wild-type plants. We obtained a list of 91 genes with commonly deregulated transcription levels in phytochrome mutants and UV-stressed wild-type plants (Fig. 9). A functional analysis of these genes is presented in Supplementary Table S4, available at *JXB* online. Within this list, we identified three genes encoding proteins involved in photosynthetic light reactions that were highly upregulated in both phytochrome mutants and in Col-0 after UV treatment. These were two light-harvesting complex PSII proteins (LHCB4.2 and LHB1B1) and PSBO1 (PSII OEC1). The expression levels of genes responsible for hormonal signalling, especially in ABA- and auxin-dependent pathways, were also deregulated in UV-triggered plants in a phyA- and phyB-dependent manner. The ABA signalling pathway component SOT16 (sulfotransferase 16) was induced in phytochrome mutants and after UV treatment, while the negative regulator of ABA signalling AIP2 (ABI3-interacting protein 2) was suppressed in *phyA* and *phyB* but elevated in UV-treated plants (Supplementary Table S4). Moreover, two genes encoding auxin-responsive proteins - SAUR-like auxin-responsive protein and AIR9 (auxin-induced in root cultures 9) - were commonly deregulated in *phy* mutants and in UV-treated Col-0. We also found that an enzyme involved in vitamin B6 biosynthesis, PDX1.1 (pyridoxine biosynthesis 1.1), was overexpressed in UV-triggered plants and in both *phy* mutants. The expression level of *VTC4* (l-galactose-1-phosphate phosphatase) involved in ascorbate biosynthesis was upregulated after UV treatment but inhibited in *phyA* and *phyB*. Furthermore, 10 transcription factors from different families were present in our list (Supplementary Table S4). Two belonging to an ethylene response factor subfamily, RAP2.1 (related to AP2 1) and RAP2.6 (related to AP2 6), were differently regulated in phytochrome mutants and UV-triggered wild-type plants. We also found that the expression levels of two genes encoding calmodulin-binding proteins - IQD14 (IQ-DOMAIN 14) and IQD30 (IQ-DOMAIN 30) - were deregulated in UV-stressed wild-type plants in a phyA- and phyB-dependent manner. These results show novel genes that may be involved in phyA- and phyB-dependent signalling pathways in response to UV. Their functional diversity casts a new light on metabolic, signalling, and molecular interactions in which these phytochromes are engaged.

## Discussion

In their natural habitat, plants are constantly exposed to various stresses. The speed and accuracy of environmental signal perception as well as appropriate responses at the molecular and cellular levels underlie plant adaptation processes and enable stress tolerance. This is essential for plants’ survival, proper growth and development, and their Darwinian fitness (Külheim *et al.*, 2002). One of the most important factor governing plant growth and development is light. The mechanisms involved in light perception and signalling are predominantly dependent on the presence of proper photoreceptors and chloroplast retrograde signalling. Among these, phytochromes receive strong attention, since they were proved to regulate different developmental plant processes. Depending on light conditions, the physiological reactions can be regulated antagonistically or synergistically by different phytochromes. phyA and phyB are examples of such a regulatory phenomenon. The rapid migration of phyA to the nucleus is promoted by very low fluences of red- and far-red light, whereas the nuclear import of phyB is activated by red light and inhibited by far-red light (Karpiński *et al.*, 2003; Schepens *et al.*, 2004; Franklin and Quail, 2010).

In this study, we characterized photosynthetic activity in phytochrome-deficient mutants *phyA*, *phyB*, and *phyAB*. The inhibition of CO_2_ assimilation rate and stomatal conductance in *phyB* and *phyAB* were associated with lower stomatal density (Figs 2 and 3, Supplementary Table S1). The role of phyB in CO_2_ uptake, transpiration rate, and stomata development has been demonstrated elsewhere (Boccalandro *et al.*, 2009; Casson and Hetherington, 2010). Nevertheless, an additional role of phyA, in combination with phyB, as a positive regulator of gas exchange efficiency, cannot be neglected, since the decrease in CO_2_ assimilation, stomatal conductance, and plant growth retardation were most visible in double *phyAB* mutant (Figs 2 and 3, Supplementary Table S1). Moreover, the inhibition of photosynthetic activity was observed in *phyB* (Fig. 7). It was not related to the changes in the total absorption area of chloroplasts (Supplementary Table S1). However, it seemed to be the result of decreased levels of chlorophylls and carotenoids (Table 1). A positive influence of phyB on chlorophyll biosynthesis has recently been shown in rice (Zhao *et al.*, 2013). Indeed, in their active forms, phyA and phyB bind to PIFs, regulating chlorophyll biosynthetic pathways (Moon *et al.*, 2008). Here, we showed that in *Arabidopsis* phyB has a positive involvement not only in chlorophyll biosynthesis but also in other photosynthetic pigments. To the best of our knowledge, our study is the first report of phyB involvement in the positive regulation of carotene concentration. Lower content of photosynthetic pigments in *phyB* was accompanied by strong upregulation of genes encoding subunits of light-harvesting complexes: LHCB4.2 and LHCB1B (Supplementary Table S4). This may suggest the attempt of *phyB* plants to compensate decreased photosynthetic efficiency by overexpressing photosynthetic pigment-binding proteins. Taking together, it can be concluded that phytochromes, mainly phyB, or phyB in interaction with phyA, positively affect photosynthetic efficiency in *Arabidopsis.*

PCD, which is a genetically controlled cellular self-destruction mechanism in all eukaryotic organisms, can be induced either as a part of normal development or in response to various stress factors (de Pinto *et al.*, 2012; Nawkar *et al.*, 2013). We observed that neither phyA nor phyB was involved in cell death regulation in the absence of stress factor (Fig. 4). In contrast to the control conditions, UV-C radiation enhanced cell death in *phyB* and *phyAB* mutants compared with wild-type plants, as indicated by ion leakage measurements and staining of dead cells with trypan blue. We showed for the first time that phyB acts as a positive regulator of plant acclimation to UV stress (Fig. 4). Moreover, induced cell death in *phyAB* may indicate that phyA also plays some role in PCD regulation. In another study, UV-B radiation was demonstrated to improve phyB-mediated cotyledon opening during *Arabidopsis* de-etiolation (Boccalandro *et al.*, 2001). These results suggest that phyB and phyA participate in signal transduction in response to UV stress.

A rapid increase in ROS production plays a key role in plant PCD induction during stress (Overmyer *et al.*, 2003; Gill and Tuteja, 2010; de Pinto *et al*, 2012). *lsd1* mutant used in our study as a control of UV-triggered PCD (Wituszyńska *et al.*, 2015) exhibited O_2_^.^ˉ and H_2_O_2_ overproduction, and runaway cell death (Figs 1 and 4). Surprisingly, the content of O_2_^.^ˉ and H_2_O_2_ in *phyB* and *phyAB* was lower before and after UV stress in comparison with wild-type plants. A decreased content of O_2_^.^ˉ and H_2_O_2_ in *phyB* and *phyAB* before stress corresponded with the lower activity of SOD, CAT, and APX enzymes (Fig. 5). Thus, decreased ROS levels in *phyB* were not caused by higher scavenging enzymes activities. One of the reason for this phenomenon could be that the defence system in *phyB* is slightly activated in non-stress conditions, as seen by the higher SA levels in this mutant. After UV-C, all tested phytochrome mutants demonstrated lower H_2_O_2_, which corresponded with decreased antioxidative enzyme activities, mostly for *phyB*. Our results are the first indication that phyB alone and in cooperation with phyA can positively regulate ROS generation by control of the antioxidative enzymatic system. However, decreased ROS accumulation in *phyB* and *phyAB* did not restrain PCD progression in these genotypes. This fact may suggest that phyB-dependent ROS production is not a key factor involved in PCD. Therefore, ROS regulation by phytochromes is rather a part of a more subtle stress signalling during phyB protection towards UV-C stress. However, more studies need to be performed to confirm this hypothesis.

Our experiments also showed accumulation of SA in *phyB* before UV stress, which indicates that phyB can act as a negative regulator of SA synthesis in non-stress conditions. However, during UV stress, the SA concentration was significantly lower in all *phy* mutants in comparison with the wild-type plants. In this context, our results imply the existence of some unknown mechanism that leads to PCD during UV-C stress in phyA- or phyB-deficient mutants, in which H_2_O_2_/SA are less important than in most already known PCD-dependent pathways (de Pinto *et al.*, 2012). Taken together, these data also demonstrate that phyB plays an important role in the regulation of UV-C-induced PCD, but independently from or with minor involvement of H_2_O_2_ and SA signalling.

UV-C radiation has been shown to negatively affect PSII efficiency (Wituszyńska *et al.*, 2015). It has been demonstrated that photosynthetic electron transport is impaired by UV, operating within the range of 250–260nm (for a review, see Vass *et al.*, 2005). To analyse more precisely the role of phyA and phyB in PSII functioning after UV stress, we performed the OJIP test. Elevated flux ratios for ABS/RC, TRo/RC, ETo/RC, and Dlo/RC were indicators of the plants’ higher sensitivity to drought (Wang *et al.*, 2012), salinity (Mehta *et al.*, 2010), cold (Liang *et al.*, 2007), high temperature (Mathur *et al.*, 2011), and combined salt and heat stress (Mathur *et al.*, 2013). The OJIP results for *phyB* plants suggested that UV-C negatively affected the maximum quantum efficiency of PSII (Phi_Po) and OEC activity (*F*_V_/*F*_0_) (Fig. 7B), but also the antenna structure and fate of absorbed photons. The increase in ABS/RC and DIo/RC in *phyB* (Fig. 7B) indicated inactivation of PSII centres after UV-C exposure. This effect was also observed in high temperature, ozone, and osmotic stress studies (Bussotti *et al.*, 2011; Kalaji *et al.*, 2011; Mathur *et al.*, 2011). All of these data suggest that the photochemical reactions were disturbed in *phyB*. Because of the UV-C-triggered damage within the photosynthetic apparatus (Wituszyńska *et al.*, 2015), *phyB* was unable to use energy, harvested by light-harvesting complexes, for photochemical reactions. This could lead to photoinhibition and cell death, but was not connected to increased production of ROS compared with the wild type.

NPQ in leaves consists of three components: state transition quenching (qT), energy dependent quenching (qE), and photoinhibitory quenching (qI). qT is most important in leaves being exposed to low light levels. In non-stressed moderate to saturating light conditions, the major component of NPQ is qE, which is associated with the V+A+Z cycle. This protective mechanism is triggered by light-driven acidiﬁcation of the thylakoid lumen, which induces the enzymatic conversion of xanthophyll V to and Z, and the protonation of PsbS protein in the thylakoid membranes (Müller *et al.*, 2001). V+A+Z cycle together with another cycle, employing lutein (lutein epoxide cycle), is involved in the thermal dissipation of excitation energy in *Arabidopsis* (Jahns and Holzwarth, 2012). In plants exposed to severe stress restricting the consumption of reductants produced by photosynthetic electron transport, qI becomes a more significant component of NPQ (Baker, 2008). Both *phyB* and *phyAB* mutants demonstrated lower NPQ values after UV exposure. However, the de-epoxidation state in these mutants was higher after UV exposure, indicating a disturbance in photosynthetic electron transport and denoting higher susceptibility towards photoinhibition. This observation may indicate that other NPQ components such as the lutein epoxide cycle or qI were less pronounced in *phyA* and *phyAB*. Nevertheless, to confirm this presumption, further studies need to be performed.

It has been shown that chlorophylls and carotenoids associated with light-harvesting complexes can be damaged by various stress factors, resulting in reduced light-absorbing efficiency of both PSI and PSII, and hence decreased photosynthetic capacity (Vass *et al.*, 2005; Ashraf and Harris, 2013). Carotenoids play two key roles: they serve as accessory pigments in photosystems and they protect photosynthetic apparatus against ROS, mainly singlet oxygen (Ramel *et al.*, 2013). Lutein and β-carotene have been reported to participate in excess light energy dissipation and free radical quenching (García-Plazaola *et al.*, 2003, 2012), PSII protection against UV radiation (White and Jahnke, 2002), and PSI photoprotection under high light at chilling temperatures (Cazzaniga *et al.*, 2012). Reduced photosynthetic efficiency and impaired PSII photochemistry, showed mainly by *phyB* plants, could be caused by decreased amounts of chlorophylls and carotenoids, but before UV-C treatment (Table 1). The significant reduction in photoprotective β-carotene content in *phyB* might also be responsible for its higher susceptibility to UV-C-induced cell death (Table 1). It has been demonstrated that severe oxidative damage in *hy1* mutant, which is defective in chromophore biosynthesis and thus severely deficient in phytochrome activities, was connected to a decrease in chlorophyll content and carotenoid/flavonoid metabolism and downregulation of antioxidant defences. Moreover, maximum expression of *HY1* in wild-type *Arabidopsis* was observed following UV-C irradiation (Xie *et al.*, 2012). These results are consistent with our data and indicate the important role of phytochromes in UV-C stress protection and signalling. Moreover, the chlorophyll *a*/*b* ratio, which is an indicator of the PSII/PSI ratio (Pfannschmidt *et al.*, 2001), was significantly reduced in all untreated phytochrome mutants (Fig. 8A), suggesting that the number of PSII complexes in these mutants was smaller compared with the wild-type plants.

It cannot be excluded that significant developmental changes in *phyB* and *phyAB* mutants (Fig. 1) influenced their photosynthetic parameters and response to UV stress. However, it has been shown recently that pre-illumination of leaves with red light (perceived by phytochromes) has the potential to inhibit the effect of UV radiation on photosynthesis and the activity of PSII. The *Arabidopsis hy2* mutant, which has reduced synthesis of phytochrome B chromophore, has been demonstrated to have decreased resistance of PSII to UV compared with the wild-type plants (Kreslavski *et al.*, 2013). This result is consisted with our data and shows that increased resistance of photosynthetic apparatus to UV radiation involves phyB.

Expression meta-analysis revealed 91 transcripts that were commonly deregulated in *phyA* and *phyB*, and in wild-type plants after UV treatment (Fig. 9, Supplementary Table S4). High upregulation of genes encoding light-harvesting PSII complex protein and PSII OEC protein might be caused by the attempt of plants to re-synthesize compounds crucial for photosynthetic apparatus, compensating effects of photosynthetic pigments loss after UV treatment. These phyA- and phyB-dependently expressed proteins can be considered as the most UV-susceptible photosynthetic apparatus components. The overexpression of gene encoding PDX1.1 (pyridoxine biosynthesis 1.1), the enzyme involved in vitamin B6 biosynthesis, was common for UV-triggered plants and both *phy* mutants. Recent studies have demonstrated that vitamin B6 in *Arabidopsis* participates in the resistance to abiotic stresses (Havaux *et al.*, 2009; Vanderschuren *et al.*, 2013). The lower expression level of gene encoding ascorbate biosynthesis enzyme VTC4 is consistent with decreased APX activity, especially in *phyB*. We also found two transcription factors belonging to the ethylene response factor subfamily, RAP2.1 and RAP2.6, that were differently regulated in phytochrome mutants and UV-triggered wild-type plants. Both of these have been shown to play an important role in defence responses to various abiotic stresses (Dong and Liu, 2010; Zhu *et al.*, 2010). Two members of the IQD family of proteins that bind calmodulin were downregulated in *phy* mutants and in the UV-treated wild type. This suggests that some involvement of calcium signalling is common for phytochrome- and UV-dependent signalling. Furthermore, we identified other genes whose products are involved in the phyA- and phyB-regulated pathways during the response to UV radiation such as: protein kinases (MPK2), proteins involved in ABA, auxin, and calcium signalling, and transcription factors from the MYB, zinc finger, and bZIP families.

## Conclusions

The results presented in this work indicate an important role of phytochromes in the control of light-mediated photosynthetic activity and UV-C-induced PCD. phyB and phyA seem to be complex regulators of photosynthesis, starting from the gene expression level - by adjusting PSII photochemistry and ROS/SA signalling level - by to photosynthesis efficiency determined at the whole-plant level. Our results shows that phyB is a positive regulator of photochemical reactions in PSII and photosynthetic acclimatory responses to UV-C-induced stress. However, an auxiliary/complementary effect of phyA as the interacting partner of phyB is also evident. Because UV has been shown to induce damage of PSII and entire chloroplasts similar to that observed by exposure to excess light (Ohnishi *et al.*, 2005), we suggest that the processes extinguishing excessive energy and preventing subsequent PCD in response to impaired photosynthetic electron transport depend on phyB and phyA.

## Supplementary data

Supplementary data are available at *JXB* online.

Supplementary Fig. S1. Cell death, expressed as the ion leakage, determined for UV-treated plants with dose 200 mJ cm^–2^.

Supplementary Fig. S2. Hydrogen peroxide concentration visualized for untreated and UV-treated plants, 96h after UV exposure (100 mJ cm^–2^) using DAB staining.

Supplementary Fig. S3. Superoxide visualized in UV-treated plants, 96h after UV exposure (100 mJ cm^–2^) using NBT staining.

Supplementary Table S1. Dry weight, stomatal density, and chlorophyll autofluorescence of wild-type and phytochrome mutants.

Supplementary Table S2. Statistical analysis of gas exchange and chlorophyll *a* fluorescence parameters in response to different light intensities or different CO_2_ concentrations.

Supplementary Table S3. Statistical analysis of chlorophyll fluorescence parameters measured via the OJIP test.

Supplementary Table S4. Expression characteristics of genes commonly regulated in *phyA* and *phyB* mutants, and in wild-type plants after UV exposure.

Supplementary Data

## Abbreviations:

A: antheraxanthin
ABA: abscisic acid
ci: intercellular CO_2_ concentration
DAB: 3,3’-diaminobenzidine
NBT: nitro blue tetrazolium
NPQ: non-photochemical quenching
PAR: photosynthetically active radiation
PCD: programmed cell death
PS: photosystem
OEC: oxygen-evolving complex
RC: reaction centre
ROS: reactive oxygen species
SA: salicylic acid
V: violaxanthin
Z: zeaxanthin.

## References

[CIT0001] Aebi H 1984 Catalase in vitro. Methods in Enzymology 105, 121–126. 6727660 10.1016/s0076-6879(84)05016-3

[CIT0002] Alscher RG Erturk N Heath LS 2002 Role of superoxide dismutases (SODs) in controlling oxidative stress in plants. Journal of Experimental Botany 53, 1331–1341. 11997379

[CIT0003] Ashraf M Harris PJC 2013 Photosynthesis under stressful environments: An overview. Photosynthetica 51, 163–190.

[CIT0004] Baker NR 2008 Chlorophyll fluorescence: a probe of photosynthesis in vivo. Annual review of plant biology 59, 89–113. 10.1146/annurev.arplant.59.032607.092759 18444897

[CIT0005] Balestrazzi A Locato V Bottone M De Gara L Biggiogera M Pellicciari C Botti S Di Gesù D Donà M Carbonera D 2010 Response to UV-C radiation in topo I-deficient carrot cells with low ascorbate levels. Journal of Experimental Botany 61, 575–85. 19917599 10.1093/jxb/erp323

[CIT0006] Beauchamp C Fridovich I 1971 Superoxide dismutase: improved assays and an assay applicable to acrylamide gels. Analytical Biochemistry 44, 276–287. 4943714 10.1016/0003-2697(71)90370-8

[CIT0007] Bilger W Björkman O 1990 Role of the xanthophyll cycle in photoprotection elucidated by measurements of light-induced absorbance changes, fluorescence and photosynthesis in leaves of Hedera canariensis. Photosynthesis Research 25, 173–85. 24420348 10.1007/BF00033159

[CIT0008] Boccalandro HE Mazza CA Mazzella MA Casal JJ Ballar′e CL 2001 Ultraviolet B radiation enhances a phytochrome-B-mediated photomorphogenic response in *Arabidopsis* . Plant Physiology 126, 780–788. 11402206 10.1104/pp.126.2.780 PMC111168

[CIT0009] Boccalandro HE Rugnone ML Moreno JE Ploschuk EL Serna L Yanovsky MJ Casal JJ 2009 Phytochrome B enhances photosynthesis at the expenses of water-use efficiency in *Arabidopsis* . Plant Physiology 150, 1083–1092. 19363093 10.1104/pp.109.135509 PMC2689964

[CIT0010] Boggs JZ Loewy K Bibee K Heschel MS 2010 Phytochromes influence stomatal conductance plasticity in *Arabidopsis thaliana* . Plant Growth Regulation 60, 77–81.

[CIT0011] Bussotti F Desotgiu R Cascio C Pollastrini M Gravano E Gerosa G Strasser RJ 2011 Ozone stress in woody plants assessed with chlorophyll *a* fluorescence. A critical reassessment of existing data. Environmental & Experimental Botany 73, 19–30.

[CIT0012] Caemmerer S Farquhar GD 1981 Some relationships between the biochemistry of photosynthesis and the gas exchange of leaves. Planta 153, 376–87. 24276943 10.1007/BF00384257

[CIT0013] Casal JJ 2013 Photoreceptor signaling networks in plant responses to shade. Annual Review of Plant Biology 64, 403–427. 10.1146/annurev-arplant-050312-120221 23373700

[CIT0014] Casson SA Franklin KA Gray JE Grierson CS Whitelam GC Hetherington AM 2009 Phytochrome B and PIF4 regulate stomatal development in response to light quantity. Current Biology 19, 229–234. 19185498 10.1016/j.cub.2008.12.046

[CIT0015] Casson SA Hetherington AM 2010 Environmental regulation of stomatal development. Current Opinion in Plant Biology 13, 90–95. 19781980 10.1016/j.pbi.2009.08.005

[CIT0016] Cazzaniga S Li Z Niyogi KK Bassi R Dall’Osto L 2012 The *Arabidopsis* szl1 mutant reveals a critical role of β-carotene in photosystem I photoprotection. Plant Physiology 159, 1745–1758. 23029671 10.1104/pp.112.201137 PMC3425210

[CIT0017] Chen M Chory J Fankhauser C 2004 Light signal transduction in higher plants. Annual Review of Genetics 38, 87–117. 10.1146/annurev.genet.38.072902.092259 15568973

[CIT0018] Chen M Chory J 2011 Phytochrome signaling mechanisms and the control of plant development. Trends in Cell Biology 11, 664–71. 21852137 10.1016/j.tcb.2011.07.002 PMC3205231

[CIT0019] Chichkova NV Kim SH Titova ES Kalkum M Morozov VS Rubtsov YP Kalinina NO Taliansky ME Vartapetian AB 2004 A plant caspase-like protease activated during the hypersensitive response. Plant Cell 16, 157–171. 14660804 10.1105/tpc.017889 PMC301402

[CIT0020] Chun L Kawakami A Christopher DA 2001 Phytochrome A mediates blue light and UV-A-dependent chloroplast gene transcription in green leaves. Plant Physiology 125, 1957–1966. 11299375 10.1104/pp.125.4.1957 PMC88851

[CIT0021] Danon A Gallois P 1998 UV-C radiation induces apoptotic-like changes in *Arabidopsis thaliana* . FEBS Letters 437, 131–136. 9804186 10.1016/s0014-5793(98)01208-3

[CIT0022] de Pinto MC Locato V de Gara L 2012 Redox regulation in plant programmed cell death. Plant Cell & Environment 35, 234–244. 10.1111/j.1365-3040.2011.02387.x 21711357

[CIT0023] Dietrich RA Richberg MH Schmidt R Dean C Dangl JL 1997 A novel zinc finger protein is encoded by the *Arabidopsis* LSD1 gene and functions as a negative regulator of plant cell death. Cell 88, 685–694. 9054508 10.1016/s0092-8674(00)81911-x

[CIT0024] Dong CJ Liu JY 2010 The *Arabidopsis* EAR-motif-containing protein RAP2.1 functions as an active transcriptional repressor to keep stress responses under tight control. BMC Plant Biology 16, 10–47. 10.1186/1471-2229-10-47 PMC2848764 20230648

[CIT0025] Force L Critchley C van Rensen JJ 2003 New fluorescence parameters for monitoring photosynthesis in plants. Photosynthesis Research 78, 17–33. 16245061 10.1023/A:1026012116709

[CIT0026] Franklin KA Quail PH 2010 Phytochrome functions in *Arabidopsis* development. Journal of Experimental Botany 61, 11–24. 19815685 10.1093/jxb/erp304 PMC2800801

[CIT0027] Franklin KA Whitelam GC 2007 Light-quality regulation of freezing tolerance in *Arabidopsis thaliana* . Nature Genetics 39, 1410–1413. 17965713 10.1038/ng.2007.3

[CIT0028] Gao C Xing D Li L Zhang L 2008 Implication of reactive oxygen species and mitochondrial dysfunction in the early stages of plant programmed cell death induced by ultraviolet-C overexposure. Planta 227, 755–767. 17972096 10.1007/s00425-007-0654-4

[CIT0029] García-Plazaola JI Esteban R Fernández-Marín B Kranner I Porcar-Castell A 2012 Thermal energy dissipation and xanthophyll cycles beyond the *Arabidopsis* model. Photosynthesis Research 113, 89–103. 22772904 10.1007/s11120-012-9760-7

[CIT0030] García-Plazaola JI Hernández A Olano M Becerril JM 2003 The operation of the lutein epoxide cycle correlates with energy dissipation. Functional Plant Biology 30, 319–324. 10.1071/FP02224 32689014

[CIT0031] Gawroński P Gorecka M Bederska M Rusaczonek A Ślesak I Kruk J Karpiński S 2013 Isochorismate synthase 1 is required for thylakoid organization, optimal plastoquinone redox status, and state transitions in *Arabidopsis thaliana* . Journal of Experimental Botany 64, 3669–3679. 23956412 10.1093/jxb/ert203 PMC3745728

[CIT0032] Genoud T Santa Cruz MT Kulisic T Sparla F Fankhauser C Metraux JP 2008 The protein phosphatase 7 regulates phytochrome signaling in *Arabidopsis* . PLoS One 3, e2699. 18628957 10.1371/journal.pone.0002699 PMC2444027

[CIT0033] Gill SS Tuteja N 2010 Reactive oxygen species and antioxidant machinery in abiotic stress tolerance in crop plants. Plant Physiology and Biochemistry 48, 909–930. 20870416 10.1016/j.plaphy.2010.08.016

[CIT0034] Graßes T Pesaresi P Schiavon F Varotto C Salamini F Jahns P Leister D 2002 The role of ΔpH-dependent dissipation of excitation energy in protecting photosystem II against light-induced damage in Arabidopsis thaliana. Plant Physiology and Biochemistry 40, 41–49.

[CIT0035] Halliday KJ Koornneef M Whitelam GC 1994 Phytochrome B and at Least One Other Phytochrome Mediate the Accelerated Flowering Response of *Arabidopsis thaliana* L. to Low Red/Far-Red Ratio. Plant Physiology 104, 1311–1315. 12232170 10.1104/pp.104.4.1311 PMC159295

[CIT0036] Havaux M Ksas B Szewczyk A Rumeau D Franck F Caffarri S Triantaphylidès Ch 2009 Vitamin B6 deficient plants display increased sensitivity to high light and photo-oxidative stress. BMC Plant Biology 9, 130. 19903353 10.1186/1471-2229-9-130 PMC2777905

[CIT0037] Idänheimo N Gauthier A Salojärvi J Siligato R Brosché M Kollist H Mähönen AP Kangasjärvi J Wrzaczek M 2014 The *Arabidopsis thaliana* cysteine-rich receptor-like kinases CRK6 and CRK7 protect against apoplastic oxidative stress. Biochemical and Biophysical Research Communications 445, 457–462. 24530916 10.1016/j.bbrc.2014.02.013

[CIT0038] Jahns P Holzwarth AR 2012 The role of the xanthophyll cycle and of lutein in photoprotection of photosystem II. Biochimica et Biophysica Acta 1817, 182–93. 21565154 10.1016/j.bbabio.2011.04.012

[CIT0039] Jeong J Choi G 2013 Phytochrome-interacting factors have both shared and distinct biological roles. Molecules and Cells 35, 371–380. 23708772 10.1007/s10059-013-0135-5 PMC3887866

[CIT0040] Kalaji HM Govindjee, Bosa K Kościelniak J Żuk-Gołaszewska K 2011 Effects of salt stress on photosystem II efficiency and CO_2_ assimilation of two Syrian barley landraces. Environmental & Experimental Botany 73, 64–72.

[CIT0041] Karpiński S Gabryś H Mateo A Karpińska B Mullineaux PM 2003 Light perception in plant disease defence signalling. Current Opinion in Plant Biology 6, 390–396. 12873535 10.1016/s1369-5266(03)00061-x

[CIT0042] Khanna R Huq E Kikis EA Al-Sady B Lanzatella C Quail PH 2004 A novel molecular recognition motif necessary for targeting photoactivated phytochrome signaling to specific basic helix-loop-helix transcription factors. The Plant Cell 16, 3033–3044. 15486100 10.1105/tpc.104.025643 PMC527196

[CIT0043] Kidokoro S Maruyama K Nakashima K Imura Y Narusaka Y Shinwari ZK Osakabe Y Fujita Y Mizoi J Shinozaki K Yamaguchi-Shinozaki K 2009 The phytochrome-interacting factor PIF7 negatively regulates DREB1 expression under circadian control in *Arabidopsis* . Plant Physiology 151, 2046–2057. 19837816 10.1104/pp.109.147033 PMC2785984

[CIT0044] Kreslavski VD Shirshikova GN Lyubimov VY Shmarev AN Boutanaev AM Kosobryukhov AA Schmitt FJ Friedrich T Allakhverdiev SI 2013 Effect of preillumination with red light on photosynthetic parameters and oxidant-/antioxidant balance in Arabidopsis thaliana in response to UV-A. Journal of Photochemistry and Photobiology B 127, 229–236. 10.1016/j.jphotobiol.2013.08.008 24080425

[CIT0045] Külheim C Agren J Jansson S 2002 Rapid Regulation of Light Harvesting and Plant Fitness in the Field. Science 297, 91–93. 12098696 10.1126/science.1072359

[CIT0046] Kuthanova A Opatrny Z Fischer L 2008 Is internucleosomal DNA fragmentation an indicator of programmed death in plant cells? Journal of Experimental Botany 59, 2233–2240. 18436542 10.1093/jxb/ern090 PMC2413271

[CIT0047] Liang Y Chen H Tang MJ Yang PF Shen SH 2007 Responses of *Jatropha curcas* seedlings to cold stress, photosynthesis-related proteins and chlorophyll fluorescence characteristics. Physiologia Plantarum 131, 508–517. 18251888 10.1111/j.1399-3054.2007.00974.x

[CIT0048] Mateo A Funck D Mühlenbock P Kular B Mullineaux PM Karpinski S 2006 Controlled levels of salicylic acid are required for optimal photosynthesis and redox homeostasis. Journal of Experimental Botany 57, 1795–1807. 16698814 10.1093/jxb/erj196

[CIT0049] Mateo A Mühlenbock P Rustérucci C Chang CCC Miszalski Z Karpinska B Parker JE Mullineaux PM Karpinski S 2004 LESION SIMULATING DISEASE 1 is required for acclimation to conditions that promote excess excitation energy. Plant Physiology 136, 2818–2830. 15347794 10.1104/pp.104.043646 PMC523344

[CIT0050] Mathur S Allakhverdiev SI Jajoo A 2011 Analysis of high temperature stress on the dynamics of antenna size and reducing side heterogeneity of Photosystem II in wheat leaves (*Triticum aestivum*). Biochimica et Biophysica Acta 1807, 22–29. 20840840 10.1016/j.bbabio.2010.09.001

[CIT0051] Mathur S Mehta P Jajoo A 2013 Effects of dual stress (high salt and high temperature) on the photochemical efficiency of wheat leaves (*Triticum aestivum*). Physiology and Molecular Biology of Plants 19, 179–188. 24431485 10.1007/s12298-012-0151-5 PMC3656182

[CIT0052] Mazzella MA Arana MV Staneloni RJ 2005 Phytochrome control of the *Arabidopsis* transcriptome anticipates seedling exposure to light. Plant Cell 17, 2507–16. 16024587 10.1105/tpc.105.034322 PMC1197430

[CIT0053] McCabe PF Levine A Meijer PJ Tapon NA Pennell RI 1997 A programmed cell death pathway activated in carrot cells cultured at low cell density. The Plant Journal 12, 267–280.

[CIT0054] McCormac AC Terry MJ 2002 Light-signalling pathways leading to the co-ordinated expression of HEMA1 and Lhcb during chloroplast development in *Arabidopsis thaliana* . The Plant Journal 32, 549–559. 12445126 10.1046/j.1365-313x.2002.01443.x

[CIT0055] Mehta P Jajoo A Mathur S Bharti S 2010 Chlorophyll *a* fluorescence study revealing effects of high salt stress on Photosystem II in wheat leaves. Plant Physiology and Biochemistry 48, 16–20. 19932973 10.1016/j.plaphy.2009.10.006

[CIT0056] Meuwly P Métraux JP 1993 Ortho-anisic acid as internal standard for the simultaneous quantitation of salicylic acid and its putative biosynthetic precursors in cucumber leaves. Analytical Biochemistry 214, 500–505. 8109740 10.1006/abio.1993.1529

[CIT0057] Möglich A Yang X Ayers RA Moffat K 2010 Structure and function of plant photoreceptors. Annual Review of Plant Biology 61, 21–47. 10.1146/annurev-arplant-042809-112259 20192744

[CIT0058] Moon J Zhu L Huq E 2008 PIF1 directly and indirectly regulates chlorophyll biosynthesis to optimize the greening process in *Arabidopsis* . PNAS Proceedings of the National Academy of Sciences 105, 9433–9438. 10.1073/pnas.0803611105 PMC2453699 18591656

[CIT0059] Mühlenbock P Plaszczyca M Plaszczyca M Mellerowicz E Karpinski S 2007 Lysigenous aerenchyma formation in Arabidopsis is controlled by LESION SIMULATING DISEASE1. The Plant Cell 19, 3819–3830. 18055613 10.1105/tpc.106.048843 PMC2174864

[CIT0060] Mühlenbock P Szechynska-Hebda M Plaszczyca M Baudo M Mateo A Mullineaux PM Parker JE Karpinska B Karpinski S 2008 Chloroplast signaling and LESION SIMULATING DISEASE1 regulate crosstalk between light acclimation and immunity in Arabidopsis. The Plant Cell 20, 2339–2356. 18790826 10.1105/tpc.108.059618 PMC2570729

[CIT0061] Müller P Li X-P Niyogi KK 2001 Non-Photochemical Quenching. A Response to Excess Light Energy. Plant Physiology 125, 1558–1566. 11299337 10.1104/pp.125.4.1558 PMC1539381

[CIT0062] Nakano Y Asada K 1981 Hydrogen peroxide is scavenged by ascorbate-specific peroxidase in spinach chloroplasts. Plant & Cell Physiology 22, 860–867.

[CIT0063] Nawkar GM Maibam P Park JH Sahi VP Lee SY Kang CH 2013 UV-induced cell death in plants. International Journal of Molecular Sciences 14, 1608–1628. 23344059 10.3390/ijms14011608 PMC3565337

[CIT0064] Ohnishi N Allakhverdiev SI Takahashi S Higashi S Watanabe M Nishiyama Y Murata N 2005 Two-step mechanism of photodamage to photosystem II, step 1 occurs at the oxygen-evolving complex and step 2 occurs at the photochemical reaction center. Biochemistry 44, 8494–8499. 15938639 10.1021/bi047518q

[CIT0065] op den Camp RGL Przybyla D Ochsenbein C Laloi C Kim C Danon A Danon A Wagner D Hideg E Göbel C Feussner I Nater M Apel K 2003 Rapid induction of distinct stress responses after the release of singlet oxygen in Arabidopsis. The Plant Cell 15, 2320–2332. 14508004 10.1105/tpc.014662 PMC197298

[CIT0066] Overmyer K Brosché M Kangasjärvi J 2003 Reactive oxygen species and hormonal control of cell death. Trends in Plant Science 8, 335–42. 12878018 10.1016/S1360-1385(03)00135-3

[CIT0067] Overmyer K Tuominen H Kettunen R Betz C Langebartels C Sandermann H Kangasjärvi J 2000 Ozone-Sensitive *Arabidopsis* rcd1 Mutant Reveals Opposite Roles for Ethylene and Jasmonate Signaling Pathways in Regulating Superoxide-Dependent Cell Death. The Plant Cell 12, 1849–1862. 11041881 10.1105/tpc.12.10.1849 PMC149124

[CIT0068] Park E Kim J Lee Y Shin J Oh E Chung WI Liu JR Choi G 2004 Degradation of phytochrome interacting factor 3 in phytochrome-mediated light signaling. Plant and Cell Physiology 45, 968–975. 15356322 10.1093/pcp/pch125

[CIT0069] Park E Park J Kim J Nagatani A Lagarias JC Choi G 2012 Phytochrome B inhibits binding of phytochrome-interacting factors to their target promoters. The Plant Journal 72, 537–546. 22849408 10.1111/j.1365-313X.2012.05114.x PMC3489987

[CIT0070] Paul ND Gwynn-Jones D 2003 Ecological roles of solar UV radiation, towards an integrated approach. Trends in Ecology & Evolution 18, 48–55.

[CIT0071] Petrov VD Van Breusegem F 2012 Hydrogen peroxide-a central hub for information flow in plant cells. AoB PLANTS pls014. 10.1093/aobpla/pls014 PMC3366437 22708052

[CIT0072] Pfannschmidt T Schütze K Brost M Oelmüller R 2001 A novel mechanism of nuclear photosynthesis gene regulation by redox signals from the chloroplast during photosystem stoichiometry adjustment. Journal of Biological Chemistry 276, 36125–36130. 11468291 10.1074/jbc.M105701200

[CIT0073] Quail P 2002 Phytochrome photosensory signalling networks. Nature Review Molecular Cell Biology 3, 85–93. 11836510 10.1038/nrm728

[CIT0074] Ramel F Mialoundama AS Havaux M 2013 Nonenzymic carotenoid oxidation and photooxidative stress signalling in plants. Journal of Experimental Botany 64, 799–805. 22915744 10.1093/jxb/ers223

[CIT0075] Reed JW Nagatani A Elich TD Fagan M Chory J 1994 Phytochrome A and Phytochrome B Have Overlapping but Distinct Functions in *Arabidopsis* Development. Plant Physiology 104, 1139–1149. 12232154 10.1104/pp.104.4.1139 PMC159274

[CIT0076] Reed JW Nagpa Pl Poole DS Furuya M Chory J 1993 Mutations in the Gene for the Red/Far-Red Light Receptor Phytochrome B Alter Cell Elongation and Physiological Responses throughout *Arabidopsis* Development. The Plant Cell 5, 147–157. 8453299 10.1105/tpc.5.2.147 PMC160258

[CIT0077] Rustérucci C Aviv DH Holt BF 3rd Dangl JL Parker JE 2001 The disease resistance signaling components EDS1 and PAD4 are essential regulators of the cell death pathway controlled by LSD1 in Arabidopsis. The Plant Cell 13, 2211–2224. 11595797 10.1105/tpc.010085 PMC139154

[CIT0078] Samuilov VD Lagunova EM Kiselevsky DB Dzyubinskaya EV Makarova YV Gusev MV 2003 Participation of chloroplasts in plant apoptosis. Bioscience Report 23, 103–117. 10.1023/a:1025576307912 14570380

[CIT0079] Schäfer E Bowler C 2002 Phytochrome-mediated photoperception and signal transduction in higher plants. EMBO Reports 3, 1042–1048. 12429614 10.1093/embo-reports/kvf222 PMC1307593

[CIT0080] Schepens I Duek P Frankhauser C 2004 Phytochrome-mediated light signalling in *Arabidopsis* . Current Opinion in Plant Biology 7, 564–569. 15337099 10.1016/j.pbi.2004.07.004

[CIT0081] Schreiber U Schliwa U Bilger W 1986 Continuous recording of photochemical and non-photochemical chlorophyll fluorescence quenching with a new type of modulation fluorometer. Photosynthesis Research 10, 51–62. 24435276 10.1007/BF00024185

[CIT0082] Stratmann J 2003 Ultraviolet-B radiation co-opts defense signaling pathways. Trends in Plant Science 8, 526–533. 14607097 10.1016/j.tplants.2003.09.011

[CIT0083] Szechyńska-Hebda M Kruk J Górecka M Karpińska B Karpiński S 2010 Evidence for Light Wavelength-Specific Photoelectrophysiological Signaling and Memory of Excess Light Episodes in *Arabidopsis* . Plant Cell 22, 2201–2218. 20639446 10.1105/tpc.109.069302 PMC2929097

[CIT0084] Thimm O Bläsing O Gibon Y Nagel A Meyer S Krüger P Selbig J Müller LA Rhee SY Stitt M 2004 MAPMAN, a user-driven tool to display genomics data sets onto diagrams of metabolic pathways and other biological processes. The Plant Journal 37, 914–939. 14996223 10.1111/j.1365-313x.2004.02016.x

[CIT0085] Trupkin SA Legris M Buchovsky AS Tolava Rivero MB Casal JJ 2014 Phytochrome B nuclear bodies respond to the low red/far-red ratio and to the reduced irradiance of canopy shade in *Arabidopsis* . Plant Physiology 165, 1698–1708. 24948827 10.1104/pp.114.242438 PMC4119049

[CIT0086] Tsang EW Bowler C Hérouart D Van Camp W Villarroel R Genetello C Van Montagu M Inzé D 1991 Differential regulation of superoxide dismutases in plants exposed to environmental stress. The Plant Cell 3, 783–792. 1820818 10.1105/tpc.3.8.783 PMC160045

[CIT0087] Vanderschuren H Boycheva S Li KT Szydlowski N Gruissem W Fitzpatrick TB 2013 Strategies for vitamin B6 biofortification of plants, a dual role as a micronutrient and a stress protectant. Frontiers in Plant Science 4, 143. 23734155 10.3389/fpls.2013.00143 PMC3659326

[CIT0088] Vass I Szilard A Sicora C 2005 Adverse effects of UV-B light on the structure and function of the photosynthetic apparatus. In: Pessarakli M, eds. Handbook of Photosynthesis. 2nd Ed, Taylor and Francis, Boca Raton, London, New York, Singapore, 827–833.

[CIT0089] Velikova V Yordanov I Edreva A 2000 Oxidative stress and some antioxidant systems in acid rain-treated bean plants. Protective role of exogenous polyamines. Plant Science 151, 59–66.

[CIT0090] Vicente MR-S Plasencia J 2011 Salicylic acid beyond defence, its role in plant growth and development. Journal of Experimental Botany 62, 3321–3338. 21357767 10.1093/jxb/err031

[CIT0091] Wang ZX Chen L Ai J Qin HY Liu YX Xu PL Jiao ZQ Zhao Y Zhang QT 2012 Photosynthesis and activity of photosystem II in response to drought stress in Amur Grape (*Vitis amurensis Rupr*.) Photosynthetica 50, 189–196.

[CIT0092] White AL Jahnke LS 2002 Contrasting effects of UV-A and UV-B on photosynthesis and photoprotection of β-carotene in two *Dunaliella* spp. Plant & Cell Physiology 43, 877–884. 12198190 10.1093/pcp/pcf105

[CIT0093] Wituszyńska W Gałązka K Rusaczonek A Vanderauwera S Van Breusegem F Karpiński S 2013a Multivariable environmental conditions promote photosynthetic adaptation potential in *Arabidopsis thaliana* . Journal of Plant Physiology 170, 548–559. 23287000 10.1016/j.jplph.2012.11.016

[CIT0094] Wituszyńska W Ślesak I Vanderauwera S Szechyńska-Hebda M Kornaś A Van Der Kelen K Mühlenbock P Karpińska B Maćkowski S Van Breusegem F Karpiński S 2013b LESION SIMULATING DISEASE1, ENHANCED DISEASE SUSCEPTIBILITY1, and PHYTOALEXIN DEFICIENT4 Conditionally Regulate Cellular Signaling Homeostasis, Photosynthesis, Water Use Efficiency, and Seed Yield in Arabidopsis Plant Physiology 161, 1795–1805. 23400705 10.1104/pp.112.208116 PMC3613456

[CIT0095] Wituszyńska W Szechyńska-Hebda M Sobczak M Rusaczonek A Kozłowska-Makulska A Witoń D Karpiński S 2015 LESION SIMULATING DISEASE 1 and ENHANCED DISEASE SUSCEPTIBILITY 1 differentially regulate UV-C induced photooxidative stress signalling and programmed cell death in Arabidopsis thaliana. Plant Cell Environ 38, 315–330. 24471507 10.1111/pce.12288

[CIT0096] Wohlgemuth H Mittelstrass K Kschieschan S Bender J Weigel HJ Overmyer K Kangasjärvi J Sandermann H Langebartels C 2002 Activation of an oxidative burst is a general feature of sensitive plants exposed to the air pollutant ozone. Plant, Cell & Environment 25, 717–726.

[CIT0097] Wonnacott TH Wonnacott RJ 1990 Introductory statistics for business and economics. Wiley, 1990.

[CIT0098] Xie YJ Xu DK Cui WT Shen WB 2012 Mutation of Arabidopsis HY1 causes UV-C hypersensitivity by impairing carotenoid and flavonoid biosynthesis and the down-regulation of antioxidant defence. Journal of Experimental Botany 63, 3869–3884. 22419743 10.1093/jxb/ers078 PMC3388838

[CIT0099] Zhao J Zhou J-J Wang Y-Y Gu J-W Xie X-Z 2013 Positive Regulation of Phytochrome B on Chlorophyll Biosynthesis and Chloroplast Development in Rice. Rice Science 20, 243–248.

[CIT0100] Zhu Q Zhang J Gao X Tong J Xiao L Li W Zhang H 2010 The *Arabidopsis* AP2/ERF transcription factor RAP2.6 participates in ABA, salt and osmotic stress responses. Gene 457, 1–12. 20193749 10.1016/j.gene.2010.02.011

