# Supplementary material for: Role of phytochromes A and B in the regulation of cell death and acclimatory responses to UV stress in Arabidopsis thaliana
Source: J Exp Bot. 2015 Sep 18;66(21):6679–95. doi: 10.1093/jxb/erv375 (PMC4623682; doi:10.1093/jxb/erv375)
Supplement: Supplementary Data [file supp_66_21_6679__index.html]

Role of phytochromes A and B in the regulation of cell death and acclimatory responses to UV stress in Arabidopsis thaliana — Role of phytochromes A and B in the regulation of cell death and acclimatory responses to UV stress in Arabidopsis thaliana — Supplementary Data 

# Role of phytochromes A and B in the regulation of cell death and acclimatory responses to UV stress in *Arabidopsis thaliana*

## Supplementary Data

Data files

- Supplementary Data - Supplementary Data
