# Supplementary material for: Role of phytochromes A and B in the regulation of cell death and acclimatory responses to UV stress in Arabidopsis thaliana
Source: J Exp Bot. 2015 Sep 18;66(21):6679–95. doi: 10.1093/jxb/erv375 (PMC4623682; doi:10.1093/jxb/erv375)
Supplement: Supplementary Data [file supp_erv375_jexbot155564_file001.pdf]

Journal of Experimental Botany

The role of phytochromes A and B in the regulation of cell death and acclimatory responses to UV stress in *Arabidopsis thaliana*

ANNA RUSACZONEK, WERONIKA CZARNOCKA, SYLWIA KACPRZAK, DAMIAN WITOŃ, IRENEUSZ ŚLESAK, MAGDALENA SZECHYŃSKA-HEBDA, PIOTR GAWROŃSKI and STANISŁAW KARPIŃSKI

SUPPLEMENTARY DATA

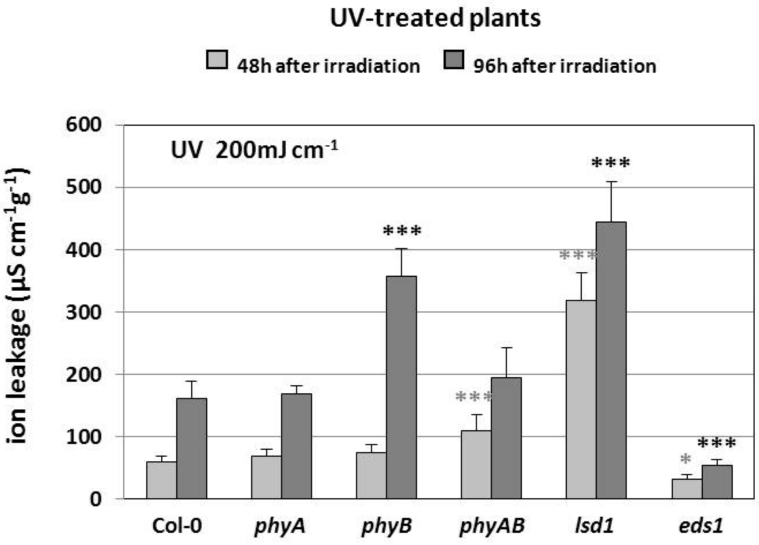

Figure S1. Cell death, expressed as the ion leakage, determined for UV-treated plants with dose 200mJ cm^{-2}. Values are means ± SD of 9-12 plants per genotype from two independent experiments (n=18-24). Asterisks indicate the significant differences from the wild-type according to the Tukey HSD test at the level of p<0.05 (*), p<0.005 (**), p<0.001 (***).

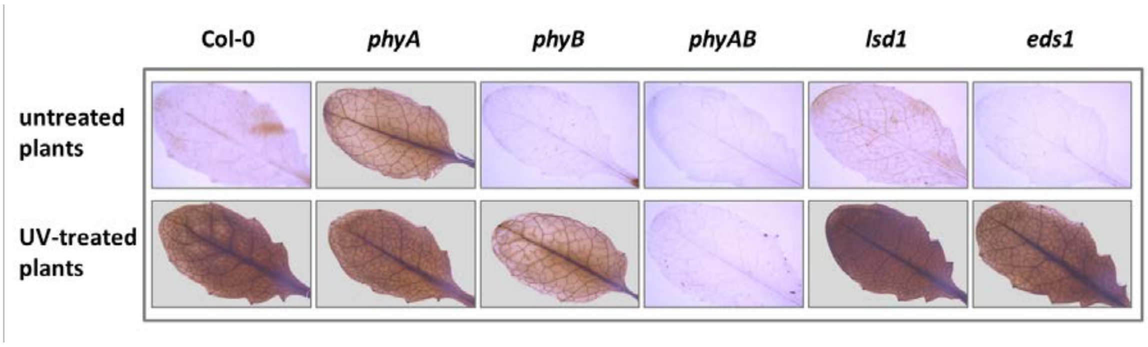

Figure S2. Hydrogen peroxide concentration visualized for untreated and UV-treated plants, 96h after UV exposure (100mJ cm^{-2}) using DAB staining.

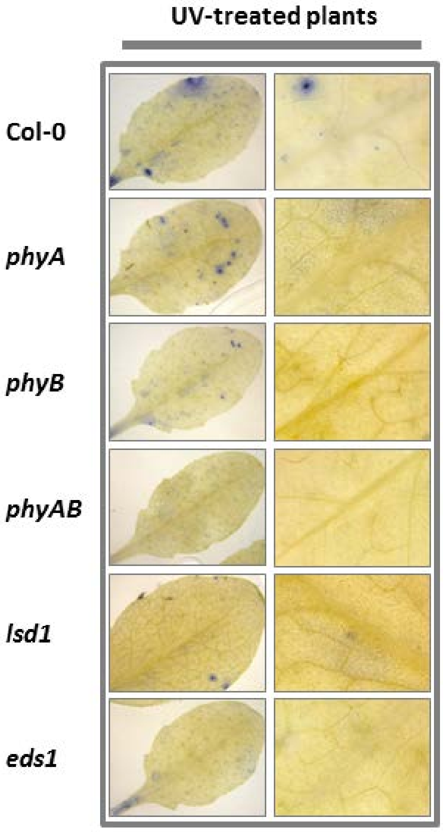

Figure S3. Superoxide visualized in UV-treated plants, 96h after UV exposure (100mJ cm^{-2}) using NBT staining.

Table S1. Dry weight, stomatal density and chlorophyll autofluorescence of wild-type and phytochrome mutants. *Arabidopsis thaliana* ecotype Col-0 (wild type) and phytochrome mutants in Columbia background. Values are means ± SD of 9-12 plants per genotype from two independent experiments (n=18-24). Asterisks indicate the significant differences in comparison to Col-0 according to the Tukey HSD test at the level p<0.05 (*), p<0.005 (**), p<0.001 (***). Different letters indicate a significant difference at p<0.05. Fluorescence distribution of the chloroplasts was calculated from the total area of the image with LSM710 ZEN 2009b software (Carl Zeiss). Bar for microscopic images, 20µm.

	**Col-0**	***phyA***	***phyB***	***phyAB***
**Dry weight [mg g^{-1} FW]**	72.0 ± 5.42 a	68.8 ± 5.17 a	64.5 ± 4.85 a	38.8 ± 2.91 *** b
**Stomatal density [no mm^{-2}]**	227 ± 32.3 a	220 ± 28.2 a	123 ± 22.1 *** b	165 ± 37.3 *** c
**Chloroplasts autofluorescence**	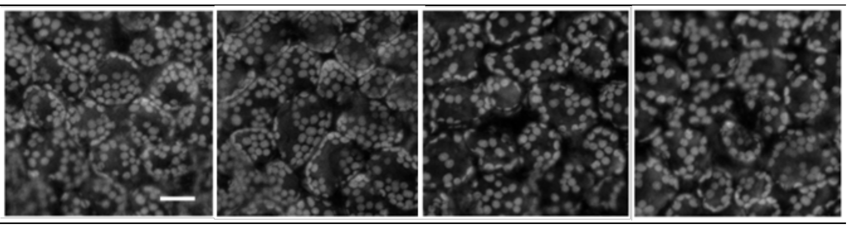			
**Mean intensity of autofluorescence**	40.9 ± 8.8	41.5 ± 4.4	40.2 ± 7.0	38.2 ± 1.3

Table S2. Statistical analysis of gas exchange and chlorophyll a fluorescent parameters in response to different light intensities (PAR) and in response to different CO_2 concentrations (ci). Different letters indicate a significant difference at p<0.05 (Tukey's test).

genotype	Assimilation Rate		GH_2O		qN		NPQ		ΦPSII	
	PAR	ci	PAR	ci	PAR	ci	PAR	ci	PAR	ci
phya	a	a	a	a	a	a	a	a	a	a
phyb	b	b	b	b	b	b	b	a	a	b
phyab	c	c	c	c	b	c	b	b	a	b

Table S3. Statistical analysis of chlorophyll fluorescence parameters measured via OJIP test in untreated plants (NT), and 96h after UV radiation with dose 100 mJ cm^{-2}. Asterisks indicate the significant differences in comparison to Col-0 according to the Tukey HSD test at the level p<0.05 (*), p<0.005 (**), p<0.001 (***) or non-significant (ns).

genotype	Vj	Fv/Fo	Mo	Phi_Po	Psi_o	Phi_Eo	Phi_Do	ABS/RC	TRo/RC	ETo/RC	DIo/RC
**NT**											
phyA	*	ns	ns	ns	*	ns	ns	***	***	***	*
phyB	**	***	***	***	**	***	***	***	***	*	***
phyAB	ns	ns	***	ns	ns	ns	ns	***	***	**	ns
**96h after UV-C radiation**											
phyA	ns	***	ns	*	*	ns	*	ns	ns	ns	ns
phyB	ns	***	**	***	***	ns	***	**	ns	ns	**
phyAB	ns	***	***	***	***	ns	***	***	***	ns	***

Table S4. Expression characteristics of 91 genes commonly regulated in *phyA* and *phyB* mutants, and in Col-0 wild-type plants after UV exposure. Data present fold change (FC) defined as difference in expression in the mutant relative to the wild-type or as difference in expression in UV-treated plants when compared to the untreated. Colors of cells range from saturated red (up-regulation) to saturated green (down-regulation).

AGI	Gene	FC Col-0 non- vs. UV-treated	FC *phyA* vs. Col-0	FC *phyB* vs. Col-0
**Light reactions**				
AT3G08940	LHCB4.2 (light harvesting complex photosystem II protein)	385	1530	1270
AT2G34430	LHB1B1 (light-harvesting chlorophyll protein complex II subunit B1)	1730	1810	1270
AT5G66570	PSBO1 (PS II oxygen-evolving complex 1)	678	1340	1230
**Glycolysis**				
AT2G22480	PFK5 (6-phosphofructokinase)	-17	-266	-281
**Tricarboxylic acid (TCA) cycle**				
AT1G59900	E1 ALPHA (pyruvate dehydrogenase complex E1 alpha subunit)	-124	-632	-790
**Cell wall modification**				
AT5G44480	DUR (UDP-arabinose 4-epimerase 4)	-20	-127	-116
AT1G26770	EXP10 (expansin A10)	59	143	310
**Lipid metabolism**				
AT1G04220	KCS2 (3-ketoacyl-CoA synthase 2)	220	-279	-215
AT3G01570	oleosin 5	-24	524	720
**Amino acid metabolism**				
AT3G06850	BCE2 (dihydrolipoamide branched chain acyltransferase)	-608	-354	-909
**Secondary metabolism**				
AT1G74100	SOT16 (sulfotransferase 16)	250	289	424
AT2G39980	HXXXD-type acyl-transferase-like protein	-284	813	904
**Hormone metabolism**				
AT5G20910	AIP2 (ABI3-interactiong protein 2)	96	-53	-106
AT5G53590	SAUR-like auxin-responsive protein	-95	88	191
AT2G34680	AIR9 (auxin-induced in root cultures 9)	-106	-518	-697
**Co-factors and vitamines metabolism**				
AT2G38230	PDX1.1 (pyridoxine biosynthesis 1.1)	101	543	208
**Stress response**				
AT2G19970	CAP (Cysteine-rich secretory proteins, Antigen 5 protein)	-36	-237	-246
AT1G11000	MLO4 (mildew resistance locus o)	-34	-139	-136
AT1G44160	HSP40/DnaJ peptide-binding protein	-18	-46	-56
**Redox status**				
AT3G02870	VTC4 (L-galactose-1-phosphate phosphatase)	32	-42	-57
**Nucleotide metabolism**				
AT2G16570	ASE1 (amidophosphoribosyltransferase 1)	20	64	43
AT3G07800	thymidine kinase	145	1640	205
AT5G20070	NUDX19 (nudix hydrolase 19)	130	-115	-98
**Biodegradation of xenobiotics**				
AT2G43430	GLX2-1 (glyoxalase 2-1)	-55	-210	-250
**C1-metabolism**				
AT5G14780	FDH (formate dehydrogenase)	-824	-1410	-727
**Miscellaneous enzyme families**				
AT2G32520	dienelactone hydrolase family protein	-35	231	130
AT2G37585	glycosyltransferase family 14 protein	-13	-91	-114
AT1G14120	2-oxoglutarate-dependent dioxygenase, putative	126	-157	-185
AT1G12160	flavin-containing monooxygenase family protein	-27	46	82
AT1G14700	PAP3 (purple acid phosphatase 3)	125	-401	-357
AT2G27190	PAP12 (purple acid phosphatase 12)	-37	-382	-301
AT1G08500	ENODL18 (early nodulin-like protein 18)	-245	-663	-361
AT3G26770	NAD(P)-binding Rossmann-fold superfamily protein	-13	84	164

**Regulation of transcription**				
AT1G46768	RAP2.1 (related to AP2 1)	-36	36	56
AT1G43160	RAP2.6 (related to AP2 6)	111	-72	-51
AT1G03790	SOM (CCCH-type zinc finger protein SOMNUS)	-150	592	737
AT5G58620	zinc finger (CCCH-type) family protein	-18	37	40
AT5G59780	MYB59 (MYB domain protein 59)	-185	-384	-286
AT1G70000	myb-like transcription factor	89	84	77
AT5G15830	bZIP3 (basic leucine-zipper 3)	-70	-500	-416
AT1G76010	Alba DNA/RNA-binding protein	-159	696	1050
AT1G10200	WLIM1 (transcription factor lim1)	-229	-428	-371
AT1G20220	Alba DNA/RNA-binding protein	28	73	64
**DNA synthesis**				
AT2G47330	DEAD-box ATP-dependent RNA helicase 24	31	-130	-160
AT1G10160	transposable element gene	-17	156	94
AT2G02090	ETL1 (helicase domain-containing protein)	16	-32	-80
**Protein synthesis and degradation**				
AT2G31610	RPS3A (40S ribosomal protein S3)	143	1130	887
AT3G04770	RPSAb (40S ribosomal protein SA B)	36	48	41
AT1G01540	serine/threonine-protein kinase, putative	-38	-263	-265
AT1G06700	serine/threonine protein kinase, putative	-61	-306	-467
AT1G20160	ATSBT5.2 (serine-type endopeptidase)	-56	-202	-222
AT5G59090	ATSBT4.12 (serine-type endopeptidase)	-115	-1080	-1000
AT1G20850	XCP2 (xylem cysteine peptidase 2)	-133	-812	-612
AT1G27910	PUB45 (plant U-BOX 45)	9	-52	-40
AT3G19950	zinc finger (C3HC4-type RING finger) family protein	49	141	196
AT1G15100	RHA2A (ubiquitin-protein ligase/ zinc ion binding)	170	149	338
AT5G27920	F-box family protein	-45	353	334
**Signalling**				
AT1G68400	leucine-rich repeat transmembrane protein kinase, putative	-23	114	107
AT1G18840	IQD30 (calmodulin binding)	26	-100	-129
AT2G43680	IQD14 (calmodulin binding)	-46	-186	-143
AT4G17170	RABB1C (RAB GTPase homolog B1C)	-224	-916	-842
AT5G59150	RABA2D (RAB GTPase homolog A2D)	-25	-108	-131
AT1G59580	MPK2 (mitogen-activated protein kinase 2)	-67	-176	-345
**Cell organisation and cycle**				
AT1G10340	ankyrin repeat family protein	10	-28	-48
AT5G20350	TIP1 (tip growth defective 1)	-84	257	-234
AT1G47230	CYCA3;4 cyclin, putative	-18	-46	-38
AT5G35100	peptidyl-prolyl cis-trans isomerase	-77	466	465
AT1G50500	HIT1 (heat-intolerant 1)	-28	-92	-146
At5g23600	KptA family RNA 2'-phosphotransferase	-68	-215	-199
AT4G17730	SYP23 (syntaxin of plants 23)	-289	-377	-564
**Development**				
AT5G58230	MSI1 (multicopy suppressor of IRA1)	30	201	119
AT3G06790	MORF3 (multiple organellar RNA editing factor 3)	19	86	80
**Transport**				
AT1G77990	AST56 (sulfate transmembrane transporter)	9	-37	-27
AT1G79520	cation efflux family protein	38	-53	-59
**Unknown**				
AT5G49560	unknown protein	-9	53	54
AT5G11090	serine-rich protein-related	-172	622	610
AT2G21530	forkhead-associated domain-containing protein	30	-141	-53
AT2G39570	ACT domain-containing protein	-472	-604	-847
AT1G15040	glutamine amidotransferase-related	-384	-633	-752
AT1G03210	phenazine biosynthesis PhzC/PhzF family protein	-59	-106	-115
AT1G21680	unknown protein	-236	591	428
AT2G22125	unknown protein	-56	-358	-441
AT1G34220	unknown protein	28	-117	-145
AT1G21050	unknown protein	-70	189	221
AT1G19140	ubiquinone biosynthetic process	33	83	106
AT4G14000	unknown protein	18	220	264
AT2G38450	unknown protein	46	287	390
AT3G03890	FMN binding	21	-65	-62
AT2G07777	unknown protein	-14	43	34
AT1G23060	unknown protein	-55	-149	-135
AT1G03730	unknown protein	-40	159	243
